# Community assembly: alternative stable states or alternative transient states?

**DOI:** 10.1111/j.1461-0248.2011.01663.x

**Published:** 2011-10

**Authors:** Tadashi Fukami, Mifuyu Nakajima

**Affiliations:** Department of Biology, Stanford UniversityStanford, CA 94305-5020, USA

**Keywords:** Beta diversity, biodiversity, environmental gradients, historical contingency, plant-soil feedback, priority effect, spatial heterogeneity, stability, succession, transient dynamics

## Abstract

The concept of alternative stable states has long been a dominant framework for studying the influence of historical contingency in community assembly. This concept focuses on stable states, yet many real communities are kept in a transient state by disturbance, and the utility of predictions for stable states in explaining transient states remains unclear. Using a simple model of plant community assembly, we show that the conditions under which historical contingency affects community assembly can differ greatly for stable versus transient states. Differences arise because the contribution of such factors as mortality rate, environmental heterogeneity and plant-soil feedback to historical contingency changes as community assembly proceeds. We also show that transient states can last for a long time relative to immigration rate and generation time. These results argue for a conceptual shift of focus from alternative stable states to alternative transient states for understanding historical contingency in community assembly.

## Introduction

It is increasingly recognised that the species composition and diversity of ecological communities can be greatly influenced by the history of community assembly. Growing evidence indicates that the effect of biotic interactions on species abundances may depend on the order and timing of species immigration during community assembly, the phenomenon known as priority effect (e.g. Schoener 1976; Drake 1991; Almany 2003). The extent of historical contingency due to priority effect is difficult to quantify because immigration history is impossible to reconstruct in sufficient detail for most natural communities. Nevertheless, theory suggests that biotic historical effects can be substantial (Gilpin & Case 1976; Drake 1990; Law 1999; Fukami 2004b; Steiner & Leibold 2004), with profound implications for understanding and conserving species diversity. For example, priority effect can cause unexpectedly high variability in community structure, or high beta diversity *sensu*Whittaker (1960, 1972), among similar sites (Fukami 2004b; Chase 2010). Further, if historical contingency is important, restoring native diversity in degraded sites may require specific sequences of species removal and introduction to be successful (Fukami *et al.* 2005; Young *et al.* 2005; Suding & Hobbs 2009; Kardol & Wardle 2010). In this light, much research has been directed toward identifying the environmental factors that determine the importance of assembly history, such as habitat productivity (Steiner & Leibold 2004), ecosystem size (Fukami 2004a), disturbance frequency (Jiang & Patel 2008) and environmental heterogeneity (Shurin *et al.* 2004; Van Nes & Scheffer 2005).

In the effort to understand the role of historical contingency in community assembly, the concept of alternative stable states (also known as multiple stable points, multiple stable equilibria, alternative attractors, multiple domains of attraction and other similar terms) has played a dominant role as the guiding theoretical framework (e.g. Lewontin 1969; Sutherland 1974; May 1977; Peterson 1984; Drake 1991; Petraitis & Dudgeon 1999; Beisner *et al.* 2003; Schröder *et al.* 2005; Suding & Hobbs 2009). According to this concept, there can be more than one final stable state of species composition that assembling communities may approach depending on immigration history, even under the same environmental conditions and the same species pool. Once a community reaches a stable state, it cannot move to another unless heavily disturbed (Lewontin 1969; Gilpin & Case 1976; Law 1999). This concept places a special emphasis on the analysis of stable states, not necessarily because stable states characterise natural communities, but primarily because of mathematical tractability (DeAngelis & Waterhouse 1987; Hastings 2004, 2010). As long recognised since at least Cowles (1899), many real communities are in a transient, not stable, state, because disturbance keeps communities from reaching a stable state (reviewed in Pickett & White 1985).

Despite this mismatch between theory and reality, theoretical predictions about alternative stable states can be useful in understanding real communities if two further assumptions are met. One assumption (hereafter assumption 1) is that, even if natural communities are not in a stable state, theoretically predicted stable states help to explain transient communities (Chase & Leibold 2003; Didham *et al.* 2005; Schröder *et al.* 2005). In other words, transient and stable states do not differ qualitatively with respect to the conditions that make assembly history important to community structure, as measured by the level of beta diversity generated by priority effect. A second assumption (hereafter assumption 2) is that, even if assumption 1 is not always true, the transient states to which assumption 1 does not apply are so short-lived that any discrepancy between stable and transient states is of minor importance.

These assumptions are, however, only tacitly implied in most studies thus far. Given the central role that the concept of alternative stable states has played in community assembly research, surprisingly little is known about the validity of these assumptions. In this paper, we examine their validity using a simple simulation model of plant community assembly. Our results suggest that both assumptions may be easily violated. The aim of this paper is not to downplay the well-appreciated importance of studying stable states, but rather to highlight the underappreciated importance of studying transient states. For example, we show that the environmental conditions under which community assembly is particularly sensitive to historical contingency can be understood only by studying transient states directly because it is often not possible to infer transient states from stable states. More generally, we seek to provide new perspectives on community assembly in order to stimulate more research on alternative transient states, which we believe will help to advance the understanding of historical contingency in community assembly and its effect on species diversity.

We define alternative transient states as follows: communities are in alternative transient states when they have not reached a stable state, but vary in structure (e.g. species composition and diversity) and/or function (e.g. total biomass and carbon flux) because of variable immigration history and other stochastic processes, even though they have assembled under the same environmental conditions, have received the same set of species multiple times, and have undergone population dynamics over multiple generations of the species involved. This definition ensures that alternative transient states do not include obvious cases in which communities vary in composition simply because they vary in environmental conditions or species pool or because they are at an early stage of assembly where species composition is inevitably variable. Thus, our definition of alternative transient states is identical to that of alternative stable states proposed by Connell & Sousa (1983) and further articulated by Chase (2003), except that communities exhibiting alternative transient states have not reached a stable state, whereas those in alternative stable states have. Here, a community is considered stable when the locally coexisting species are permanent members of the community and are resistant to colonisation by any additional species in the region (Law 1999).

In the following sections, after describing the main model employed, we will present results that indicate that assumptions 1 and 2 can easily be violated. We will then discuss implications of the violated assumptions for understanding how the importance of historical contingency varies along environmental gradients. Because any theoretical prediction needs to be evaluated by empirical evidence, we will also discuss empirical data relevant to our simulation results. We will end by suggesting several future research directions for further improving our understanding of alternative transient states.

## Model Description

### Overview

Our model is a modification of the generalised competition model analysed by Chesson (1985), Pacala & Tilman (1994), Hurtt & Pacala (1995) and Mouquet *et al.* (2002). In our model, species are randomly chosen each year from a regional species pool. The chosen species immigrate as a small number of seeds to a local patch consisting of numerous cells that vary in habitat condition. Initially, all cells are empty. Subsequently, only one individual can establish in each cell even when multiple individuals arrive from the regional pool or from within the patch. Thus, individuals compete at the establishment stage. Of the individuals that arrive at a cell, the one that belongs to the species that best fits the environmental condition of the cell wins. Once established, individuals produce seeds once a year until they die. Individuals die with a fixed probability, and when they do, the previously occupied cells become empty and available for a new individual to establish. This process of immigration, arrival, establishment, reproduction and death is repeated for multiple years.

### Regional species pools and local patches

Regional species pools each contain 30 species, with species *i* assigned a trait value, *R*_*i*_, chosen randomly from a uniform distribution [0, 1]. Local patches consist of a linear, circular array of 2000 cells. The condition of cell *j* is defined by a value, *H*_*j*_, chosen randomly between 0 and 1 from a beta distribution, where the probability density for value *x* is proportional to: *x*^*a*−1^ (1 – *x*)^*b*−1^. In our model, we set *a*= *b* and use *h* (=1/*a*), which takes values between 0 and 1 (see below), as a measure of the spatial environmental heterogeneity (e.g. soil temperature, soil moisture, soil pH) in the patch. Larger values of *h* indicate greater heterogeneity within the patch (Mouquet *et al.* 2002). Cells are distributed randomly in the patch with respect to *H*_*j*_ values.

### Community assembly

Each year, each species in the regional species pool immigrates to the local patch with a probability *I*, equal for all species (*I*=0.05). At each cell in the local patch, species *i* arrives with the probability: 1 – exp [−(*P*_*i*_ + *F N*_*i*_)/(total number of cells, i.e. 2000)]. Here *P*_*i*_ is the number of individuals of species *i* that immigrate from the regional pool (20 individuals for species chosen that year for immigration from the regional pool, and 0 individual for all other species), *F* is fecundity (50 for all species), and *N*_*i*_ is the number of individuals of species *i* in the local patch (0 for all species in the first year, i.e. at *t*=1). When the number of cells that are assigned to receive a seed of species *i* exceeds *P*_*i*_ + *F N*_*i*_ (which rarely happens), *P*_*i*_ + *F N*_*i*_ cells are randomly selected from these cells, and a seed of the species assigned only to the selected cells.

Given this probability, there are three possibilities regarding individual establishment in each cell. First, if the cell is already occupied by an individual, that individual remains there. Second, if the cell is empty, of the species that arrive at that cell, the one with the greatest value of *C*_*ij*_ establishes. The value of *C*_*ij*_, which defines the competitive ability of species *i* at cell *j*, is given as: 1–|*H*_*j*_–*R*_*i*_| if neither cell *j*− 1 nor cell *j*+1 is already occupied by species *i*; 1–|*H*_*j*_–*R*_*i*_| + *f* if cell *j*− 1 or cell *j*+1 is already occupied by species *i*; and 1–|*H*_*j*_–*R*_*i*_| + 2*f* if both cell *j*− 1 and cell *j*+1 are already occupied by species *i*. The value of *f* is positive or negative, respectively, when the presence of conspecifics in neighbouring cells increases or decreases the competitive ability of species *i* relative to other species. A biological basis for such neighbouring effects is plant-soil feedback (Bever 2003; Eppstein & Molofsky 2007). We set *f*=0, 0.05 or 0.1 for all species and for all cells. We use positive *f* values to simulate positive feedback in our model as a mechanism of priority effect (Knowlton 1992, 2004; Bever 2003; Eppstein & Molofsky 2007; Kardol *et al.* 2007; Suding & Hobbs 2009). In some plant communities, feedback may be negative rather than positive (e.g. Kardol *et al.* 2006), and may affect individuals in the same cell rather than neighbouring cells (e.g. Bever 2003; Levine *et al.* 2006). We will discuss these and other possibilities as future research directions, but focus in this paper on positive feedback as a simple example of a source of alternative stable states. Third, if the cell is empty and no species arrives at that cell, it remains empty. After individual establishment is completed for all cells, individuals occupying a cell die with the probability, *m*. We set *m*=0.1 or 0.5 for all species.

In our model, competitive ability, *C*_*ij*_, does not directly affect fecundity or mortality, but does affect the ability to ‘fight’ for a cell, which indirectly affects fecundity and mortality. The assumption that species are identical in mortality and fecundity is also made by the neutral theory (Bell 2001; Hubbell 2001), but in our model, species are not neutral, because competitive ability, *C*_*ij*_, differs between species. Moreover, the neutral theory focuses on explaining the structure of equilibrium communities, whereas we focus on explaining the structure of transient communities.

Following these rules of immigration, arrival, establishment, reproduction and death for 1600 generations (for *t*=1600 years), we assemble 10 communities using the same set and distribution of *H*_*j*_ values in the patch under each regional pool used. Two observations confirm that communities always reach a stable state by the 1600th generation in our model. First, there is no obvious long-term change in immigration and extinction rates from the 1200th to 1600th generations, indicating that communities have entered an equilibrium state by, conservatively, the 1600th generation (see Fig. S1 in Supporting Information). Second, between the 1200th and 1600th generations, there is virtually no immigration (indicating that communities are resistant to invasion by any additional species from the regional pool) or extinction (indicating that communities have stable species composition with no species lost over time) if immigration and extinction are measured for species having more than 100 individuals in the patches, indicating that communities have reached a stable state (see Fig. S1). In contrast, communities are still in a transient state at the 60th generation, as indicated by immigration and extinction rates still changing over time (see Fig. S1). Below we mainly compare communities observed at *t*=60 and *t*=1600 as those at transient and stable states, respectively.

### Species diversity

We measure alpha diversity as the mean number of species present in a local patch (averaged over the 10 replicate communities), gamma diversity as the number of species present in one or more of the 10 patches, and beta diversity as gamma diversity divided by alpha diversity. This measure of beta diversity is the original multiplicative form of Whittaker (1960, 1972). Although other measures of beta diversity have been proposed (Koleff *et al.* 2003; Tuomisto 2010; Anderson *et al.* 2011), we use Whittaker's measure for two reasons. First, it can be interpreted as indicating the number of alternative community states observed in different patches in the region (Jost 2010; Wilsey 2010), or more precisely, the effective number of distinct local communities in the region (Jost 2007, 2010; Wilsey 2010), applicable for both transient and stable states. Thus, multiplicative beta diversity can be used as a surrogate for the effective number of alternative states, which can be used to evaluate the importance of immigration history in community structuring. Second, unlike some other measures of beta diversity, the multiplicative measure is comparable between regions even when alpha diversity is variable between regions (Jost 2010; Wilsey 2010). We note, however, that further analysis indicates that our main conclusions regarding the validity of assumptions 1 and 2 hold true when we use the additive, rather than multiplicative, measure of beta diversity, calculated as gamma diversity minus mean alpha diversity (Lande 1996; Crist *et al.* 2003; Veech & Crist 2010).

### Mortality rate, environmental heterogeneity and the strength of intra-specific feedbacks

To examine the effect of mortality rate (*m*), habitat heterogeneity (*h*) and the strength of plant-soil feedback (*f*) on the importance of historical contingency as measured by beta diversity, we run the simulation using all possible combinations (hereafter called scenarios) of the following parameter values: *m*=0.1 and 0.5; *h*=0.0125, 0.025, 0.05, 0.1, 0.2 and 0.4; and *f*=0, 0.05 and 0.1 (Fig. S2). We analyse 20 replicates (20 independently created pairs of the regional pool and local patch) to examine alpha, beta and gamma diversity for each combination of *m*, *h* and *f* values (Fig. S2).

## Model Results

### Evaluating assumption 1: are stable states and transient states comparable?

**Figure 1:**
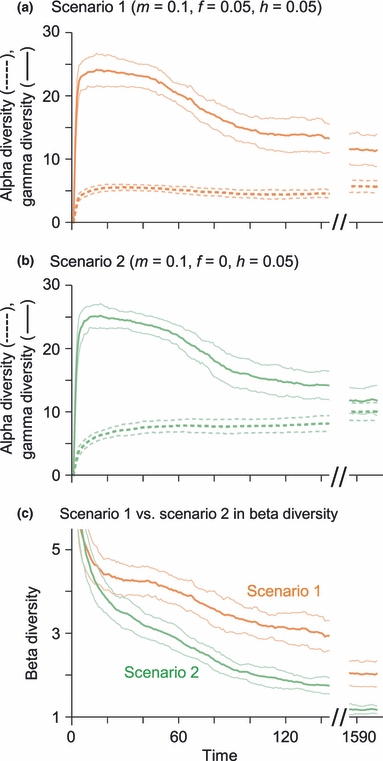
Illustrative example of community assembly where assumption 1 is valid: beta diversity is higher in one scenario (a) than in the other (b) for both transient and stable states, except at very early stages of assembly. Temporal changes in alpha, beta and gamma diversity are presented with means (dark lines) and standard deviations (pale lines). Transient dynamics (from *t*=1 – 150) and stable states (*t*=1580 – 1600) are shown. In (c), beta diversity is presented for both scenarios to facilitate comparison between them.

To investigate the validity of assumption 1, we now use several illustrative examples of simulation results. Some examples indicate that assumption 1 is sometimes valid. For instance, comparing two scenarios of community assembly, one with positive feedback and one without (Fig. 1), we find, for stable communities (i.e. at *t*=1600), that beta diversity is higher when there is positive feedback. This is an expected result: in general, when the strength of positive feedback ( *f* )= 0, there is only a single stable state that is approached by the assembled communities, but when *f*>0, alternative stable states exist, as confirmed by the fact that beta diversity is greater than 0 at *t*=1600, even when only species with more than 100 individuals in a given community are regarded as members of that community (Fig. S3; see also Figs S4–S7). In any case, except at very early stages of community assembly (until *t*= ∼20), the relative difference in the level of beta diversity between the two scenarios is the same, throughout all stages of assembly, as the eventual outcome for stable communities, despite the slow gradual decline in the absolute value of beta diversity in both scenarios (Fig. 1). Therefore, in this case, the prediction that the number of alternative states is greater in the presence of positive feedback than in its absence is consistent between stable and transient communities.

**Figure 2:**
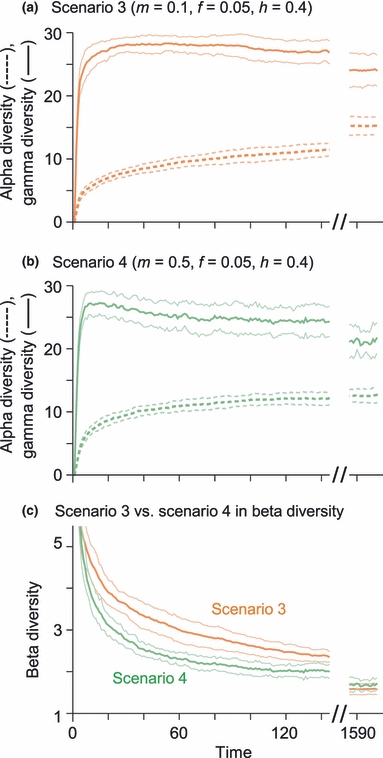
Illustrative example of community assembly where assumption 1 is violated: beta diversity differs between the scenarios during transient dynamics, but not at stable states. Symbols are as in Fig. 1.

Assumption 1 is not always valid, however. For example, comparing two scenarios, the number of alternative states can be indistinguishable for stable communities, but different in transient communities. In the example shown in Fig. 2, the two scenarios differ only in mortality rate. Beta diversity does not differ between the two scenarios for communities at a stable state, as expected from the same value of *f* shared between the scenarios. However, it continues to be different for a long time (until *t*= ∼150) during transient dynamics. Here, mortality rate determines the rate at which beta diversity approaches the final value, causing beta diversity to differ for transient, but not stable, states (Fig. 2). We will refer to these dynamics (Fig. 2c) as slow convergence of beta diversity between scenarios.

**Figure 3:**
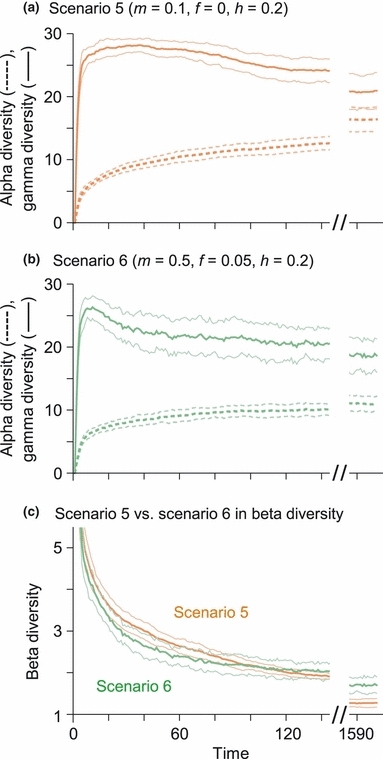
Illustrative example of community assembly where assumption 1 is violated: beta diversity differs between the scenarios at stable states, but not during transient dynamics. Symbols are as in Fig. 1.

Conversely, beta diversity is in some cases different for stable communities, but indistinguishable for transient communities. In the example shown in Fig. 3, beta diversity of stable communities is again higher in the presence than absence of positive feedback, as expected. But this difference becomes apparent only after *t*= ∼150. The two scenarios differ from each other in the values of *m* and *f*. Here the rate at which beta diversity approaches the final value (which is influenced by *m*) and the level of the final value itself (which is determined by *f*) cancel each other out for a long time in their influence on beta diversity before the eventual difference in beta diversity emerges (Fig. 3). We will refer to these dynamics (Fig. 3c) as slow divergence of beta diversity between scenarios.

**Figure 4:**
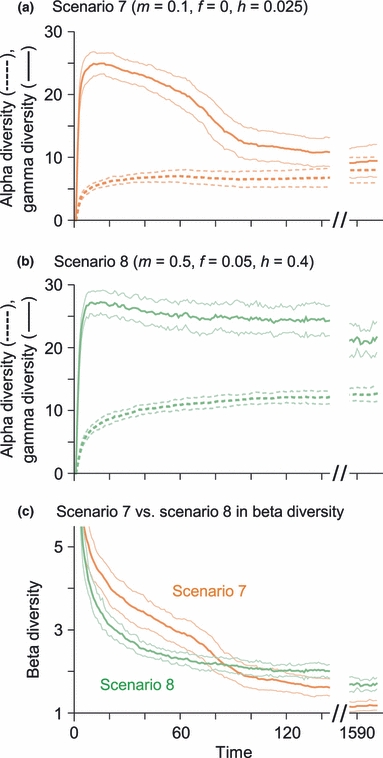
Illustrative example of community assembly where assumption 1 is violated: beta diversity shows temporal reversal between the scenarios (see text for detail). Symbols are as in Fig. 1.

The most troubling case is when beta diversity is higher in one scenario than in another for stable communities, but lower in the former scenario than in the latter for transient communities. In the example given in Fig. 4, the two scenarios differ in *h*, *m* and *f* values. The value of *h* determines the extent of initial ‘overshooting’ in alpha, gamma and beta diversity, with smaller *h* values (i.e. less heterogeneous environments) causing more extensive overshooting (Fig. 4). Because of the overshooting, small *h* values, like small *m* values, reduce the rate of the approach to the final value of beta diversity determined by the value of *f*. Consequently, the smaller *m* and *h* values in scenario 7 than in scenario 8 in Fig. 4 result in greater beta diversity until *t*= ∼70, even though beta diversity will eventually become higher in scenario 8 than in scenario 7 because of stronger positive feedbacks (i.e. larger *f* ). We will refer to these dynamics (Fig. 4c) as temporal reversal of beta diversity between scenarios.

**Figure 5:**
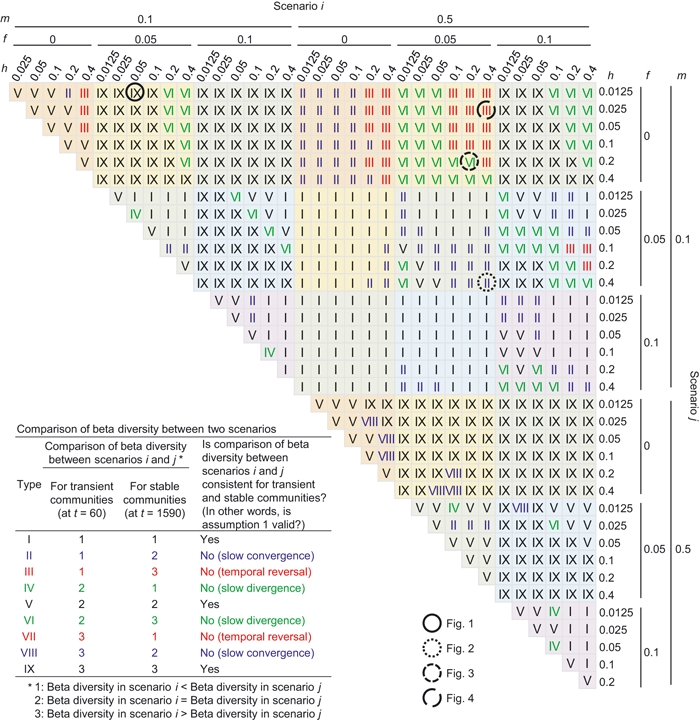
Summary of all possible pair-wise comparisons of the scenarios shown in Fig. S2.

A comprehensive pair-wise comparison of scenarios reveals that slow convergence (Fig. 2c), slow divergence (Fig. 3c) and temporal reversal (Fig. 4c) are not uncommon in the parameter space examined (Fig. 5). For example, slow convergence occurs frequently when two scenarios share the same *f*, but differ in *m*, whereas slow divergence occurs when one scenario has a higher *f* and either a higher *m* or *h* (or both) than the other. These conditions for slow divergence sometimes result in temporal reversal instead, especially when the scenario with a high *f* has a particularly high *m* or *h* (or both) relative to the other scenario. This is because strong positive plant-soil feedback (high *f*) results in an increased number of alternative stable states, whereas low mortality (low *m*) and/or low environmental heterogeneity (low *h*) result in an increased number of alternative transient states due to temporary ‘overshooting’ of gamma and beta diversity.

Additional simulations, in which 10 replicate communities are assembled using a single common immigration history (blue lines in Fig. S8), show that temporal reversal (Fig. 4c) would never occur if there was no variation between communities in immigration history (Fig. S9). Thus, these simulations reveal the importance of immigration history, relative to other sources of historical contingency (such as stochastic variation in individual establishment and mortality), in causing inconsistencies in diversity patterns between stable states and transient states.

**Figure 6:**
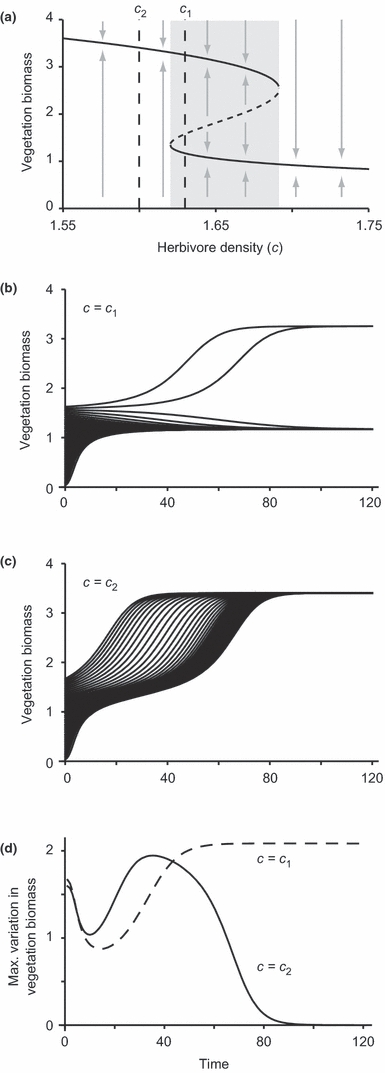
Vegetation recovery in the model defined by *dx*/*dt*= *rx* (1 – *x*/*K*) –*cx*^2^/(*x*^2^ + 1) (see text for parameter details). In (a), black line indicates stable equilibrium, dotted line indicates unstable equilibrium, and arrows indicate the direction of change in vegetation biomass under a given value of *c*. Two alternative stable states exist for a certain range of *c* (indicated by shading), as indicated by two stable equilibria for a given *c* value. In (b) and (c), trajectories of vegetation biomass recovery from 50 different initial values (i.e. 1/100, 2/100, 3/100, 4/100, … 49/100 and 50/100 of the equilibrium value) are shown for each of the two values of *c* (*c*_1_ and *c*_2_) indicated in (a). In (d), temporal changes in maximum variation in vegetation biomass between recoveries from different initial values are shown under *c*_1_ and *c*_2_.

We also point out that occurrence of temporal reversal (Fig. 4c) does not seem to depend on specific characteristics of our model. As a representative example, we use a classic grazing model of vegetation that has been extensively used for studying alternative stable states, especially with the graphical representation of the model (Fig. 6a): *dx*/*dt*= *rx*(1 − *x*/*K*) –*cx*^2^/(*x*^2^ + 1), where *r* is the per capita growth rate (we assume *r*=1), *x* is the total vegetation biomass of a plant community, *K* is the carrying capacity of total vegetation biomass and *c* is the maximum rate of grazing determined by herbivore density (Van Nes & Scheffer 2005). Using this model originally developed by Noy-Meir (1975) and May (1977), we examine temporal changes in vegetation biomass (as an aggregate property of a plant community) after biomass is reduced by pulse disturbance to less than half of the maximum level. As this model does not consider plant species composition, but just total vegetation biomass, we use between-community variation in total vegetation biomass, instead of beta diversity, as an index of the degree of historical contingency. When grazing rate *c* is 1.60, there is only one stable state (Fig. 6c), whereas when it is 1.63, there are two alternative stable states that vegetation biomass will tend to after disturbance, depending on initial biomass (Fig. 6b). When *c* is 1.60 (Fig. 6c), if biomass starts with a very low value, it will first reach a value at which the rate of biomass increase is small. Vegetation biomass will stay there for some time before complete recovery (‘ghost of equilibrium,’*sensu*Van Geest *et al.* 2007). If biomass starts above the level at which this slow change occurs, it will increase rapidly toward the stable state. This difference in transient dynamics causes transient divergence (for time= ∼10 –∼40) and then eventual convergence (completed by time= ∼90) in vegetation biomass (Fig. 6c). In contrast, when *c* is 1.63 (Fig. 6b), divergence proceeds relatively slowly. Because of this contrast in the vegetation recovery dynamics under the two values of *c*, temporal reversal in the level of biomass variation (Fig. 6d) happens (see also Van Geest *et al.* 2007; Van Nes & Scheffer 2007). We have found similar results using the two other basic models of alternative stable states studied by Van Nes & Scheffer (2005).

In summary, our results indicate that the alternative stable states concept and the predictions derived from it can be potentially highly misleading in predicting the importance of historical contingency in community assembly. For example, as apparent in our results, the number of alternative stable states may be determined solely by the strength of plant-soil feedback, whereas the number of alternative transient states may be determined not only by the strength of feedback, but also by other factors such as mortality rate and environmental heterogeneity. These factors influence the trajectory and speed of community assembly as communities approach their final stable states, affecting transient, but not stable, community states. Consequently, there can be relatively many alternative transient states even when there are few alternative stable states (e.g. scenario 7 in Fig. 4c) and vice versa (e.g. scenario 8 in Fig. 4c).

### Evaluating assumption 2: are transient states trivial?

If assumption 1 is not always valid, the next question is whether discrepancies between transient and stable patterns are trivial because they are short-lived (assumption 2). Our simulation results indicate the answer is no. Not all of the transient states that show inconsistent patterns with stable states are trivial. We have examined communities observed at *t*=60 to investigate transient states. This time scale, for example, is not trivial, for three reasons. First, more than 99.9% of individuals that are present at time *t* will have died and been replaced by new individuals by time *t*+60 even when mortality rate is relatively low (*m*=0.1). Second, virtually all species in the species pool will have had multiple chances (three times, on average) of immigration to each patch. Third, the time required for a given species to reach carrying capacity after immigration is only three generations in our simulation, so 60 generations is 20 times longer than the time needed to reach carrying capacity. Therefore, in a real plant community similar to our modelled community, if physical disturbance resets community assembly at least as frequently as every 60 years on average (e.g. major flood as disturbance to floodplain plant communities; Bellingham *et al.* 2005), predictions about the role of biotic historical contingency that depend on the assumption that communities are in stable states can be misleading in a non-trivial manner.

### Historical contingency along environmental gradients

**Figure 7:**
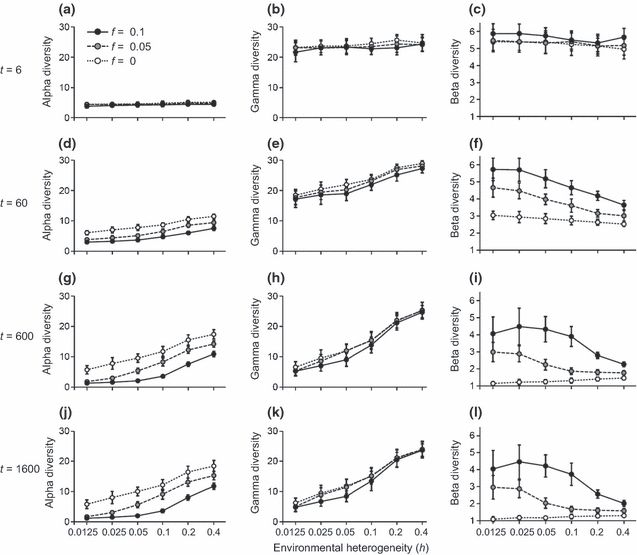
Alpha (a, d, g, j), beta (c, f, i, l) and gamma (b, e, h, k) diversity (means and standard deviations) as a function of environmental heterogeneity (*h*), positive feedback strength ( *f* ) and timing of observation (*t*). Mortality rate (*m*) is 0.1 for all cases.

Here we directly examine the issue of how the importance of assembly history varies along environmental gradients. We do this by using spatial environmental heterogeneity (*h*) as an example of a factor affecting the importance of assembly history (Fig. 7). Simulation results show that the number of alternative states, as measured by multiplicative beta diversity, can differ in the way it varies along a gradient of environmental heterogeneity, depending on whether the focus is on transient or stable states (Fig. 7c, f, i, l). For example, when positive feedback is relatively strong (*f*=0.1), beta diversity shows a hump-shaped relationship with environmental heterogeneity for stable communities (black circles in Fig. 7l), whereas it shows no effect (black circles in Fig. 7c) or a monotonic decrease (black circles in Fig. 7f) for transient communities. This difference is statistically significant (as confirmed by a Mitchell-Olds & Shaw test for the location of quadratic extreme; Oksanen *et al.* 2011; based on Mitchell-Olds & Shaw 1987). With a moderate level of positive feedback (*f*=0.05), beta diversity first declines with environmental heterogeneity and then stays constant for stable communities (grey circles in Fig. 7l), whereas it shows no effect (grey circles in Fig. 7c) or a monotonic decline with environmental heterogeneity for transient communities (grey circles in Fig. 7f). These patterns indicate that consideration of determinants of alternative transient states rather than those of alternative stable states may be more informative in order to understand when we should expect history to matter to community assembly and species diversity. Studying stable states may sometimes help to understand qualitative features of transient communities, but may not always provide quantitative predictions about transient communities.

To explain mechanistically the effects of *h* on beta diversity of stable communities, it helps to first examine how *h* affects alpha and gamma diversity. As expected, greater heterogeneity promotes alpha diversity: heterogeneity creates different ecological niches, allowing species to coexist locally (Fig. 7j). Within a given *h* value, greater positive feedback inhibits alpha diversity (Fig. 7j), as greater feedback causes greater dominance by fewer species that benefit from positive feedback, thereby excluding a greater number of species from the community. However, comparing *f*=0 and *f*=0.05 (white and grey circles in Fig. 7j), the difference in alpha diversity becomes smaller as *h* increases from 0.0125 to 0.1, indicating that priority effect reduces alpha diversity more greatly in less heterogeneous patches. On the other hand, comparing *f*=0 and *f*=0.1 (white and black circles in Fig. 7j), the difference in alpha diversity becomes larger as *h* increases from 0.0125 to 0.05. In this range of *h*, alpha diversity increases when *f*=0, whereas it stays at 1 (i.e. single-species dominance) when *f*=0.1. In the latter case, the feedback effect is so strong that a single species dominates the entire patch when *h*<0.05. Greater *h* values also promote gamma diversity, as expected (Fig. 7k). Unlike alpha diversity, however, *f* does not affect gamma diversity, indicating that any reduction in alpha diversity by *f* is compensated for by an increase in beta diversity with *f*.

Using these interpretations of alpha and gamma diversity, we can explain the response of beta diversity to environmental heterogeneity (*h*) under different strengths of positive feedback (*f*). When *f*=0.1, beta diversity increases and then decreases with *h* (black circles in Fig. 7l). The initial increase is due to dominance by a single species regardless of the *h* value, which keeps alpha diversity at 1, while gamma diversity increases with *h*. The subsequent decline in beta diversity occurs because the more heterogeneous the environment is, the more greatly the positive feedback effect is overwhelmed by environmental heterogeneity. When *f*=0.05, beta diversity is highest at *h*=0.0125 and decreases with *h* for the same reason as for when *f*=0.1 (grey circles in Fig. 7l). Unlike at *f*=0.1, beta diversity does not increase initially with *h*, because positive feedback is not strong enough to keep alpha diversity at 1. Finally, when *f*=0, beta diversity is always small regardless of *h*, as expected from the absence of positive feedbacks as a cause of alternative states (white circles in Fig. 7l).

Although these explanations make sense for stable communities, patterns that emerge in beta diversity for transient communities differ from those for stable communities. As they do at *t*=1600, both alpha and gamma diversity increase with *h* at *t*=60 (Fig. 7d, e). However, alpha diversity is greater at *t*=60 relative to *t*=1600 when *h* is small (*h*=0.0125–0.05), due to the overshooting effect discussed above, whereas it is smaller at *t*=60 relative to *t*=1600 when *h* is large (*h*=0.1–0.4), due to the slow build-up of species in communities through sequential immigration. As a result, the effect of *h* on alpha diversity is smaller, or more specifically, the slope of the increase in alpha diversity with *h* is shallower, at *t*=60 (Fig. 7d) than at *t*=1600 (Fig. 7j). Similarly, gamma diversity shows a less steep increase with *h* at *t*=60 (Fig. 7e) relative to at *t*=1600 (Fig. 7k), with the difference between gamma diversity at *t*=60 vs. *t*=1600 greater under smaller *h* values, because of the slower process of species sorting after initial community divergence. Consequently, at *t*=60, the more greatly elevated gamma under smaller *h*, combined with the lack of a rapid increase in alpha diversity with *h*, results in a monotonic decline of beta diversity with increasing *h* regardless of *f* values (Fig. 7f).

## Empirical Evidence for Inconsistency Between Stable and Transient States

Despite the difficulty in detecting historical contingency empirically (Schröder *et al.* 2005), limited evidence, mostly from highly controlled laboratory experiments, supports some of our simulation results. For example, in a microbial microcosm experiment (Fukami 2004a), variation in immigration history caused communities to assume alternative states for 20–40 generations of the species involved after all species were introduced to microcosms, depending on ecosystem size. However, communities eventually converged on a single stable state. Consequently, the effect of ecosystem size on the number of alternative states depended on the timing of observation relative to the stage of community assembly. Similarly, in another microbial experiment (Jiang & Patel 2008), the magnitude and direction of the effect of disturbance frequency (or mortality rate) on the number of alternative states (as measured by community similarity) depended greatly on the timing of observation for over 40 generations of the species involved after all species were introduced sequentially to the microcosms. Although most studies on alternative states have focused on community assembly over ecological time, another microbial experiment (Fukami *et al.* 2007) provides evidence for the long-term existence of a large number of alternative transient states over evolutionary time even though they eventually converge, with no alternative stable state. These studies provide empirical evidence that failure to consider transient dynamics severely limits our understanding of community assembly.

Evidence is scarce from field studies (Schröder *et al.* 2005), but a few ongoing long-term experiments of plant community assembly are relevant. Care is needed in interpreting field results because factors other than assembly history may have changed. That said, some studies indicate that transient states may sometimes occur only for a short time. For example, Collinge & Ray (2009) observed rapid community convergence in plant community assembly in vernal pools in California. In this experiment the high level of beta diversity experimentally imposed by manipulation of initial immigration history disappeared relatively quickly (apparent only during the first 3 years). In contrast, other studies show relatively long-lived transient states. For example, Fukami *et al.* (2005) observed slow functional community convergence during the first 9 years of experimental community assembly that was started on initially bare abandoned agricultural field. The results suggest that the communities may converge eventually, but it may take a long time for complete convergence to occur.

Additionally, empirical work on succession suggested that communities sometimes undergo ‘multiple successional pathways’ for a long time, indicating the long-term maintenance of alternative transient states despite eventual community convergence (Levin 1976; Fastie 1995; Kurkowski *et al.* 2008). Note, though, that some authors (e.g. Taylor & Chen 2010) refer to multiple successional pathways when environmental conditions vary across localities (edaphic conditions, disturbance regime, etc.). Variation in community structure due to environmental factors is different from the biotically induced variation we have focused on in this paper. The ‘state-and-transition’ models of succession (e.g. Westoby *et al.* 1989; Jackson & Bartolome 2002) are also relevant, but many of these models and empirical tests do not explicitly consider the rate of transition. More studies on the determinants of the rate at which communities approach stable states are needed to understand alternative transient states (Anderson 2007; Walker & del Moral 2009).

## Future Directions

### Other types of plant-soil feedback

The model we have used here is only a first step. For example, we have used simple positive plant-soil feedback as an easily interpretable mechanism of priority effect and alternative stable states. Recent work suggests, however, that negative plant-soil feedback may be more common at early stages of succession, whereas positive plant-soil feedback dominates at late stages (e.g. Kardol *et al.* 2006). In contrast to positive feedback, negative feedback promotes local species coexistence, but does not cause alternative stable states. Combined effects of positive and negative feedbacks on alternative transient states remain little explored. Similarly, although we have assumed that feedback effects come from neighbours, feedback may in some cases operate more locally, such that individuals that die in a given location may affect establishment of con-specifics only in that location the next year, with little effect on neighbouring locations. Future research should investigate various feedback mechanisms to better understand differences between the conditions that promote alternative transient states versus alternative stable states.

### Other types of interactions and communities

We have assumed in our model that species engage in pre-emptive competition, as opposed to dominance competition (*sensu*Amarasekare *et al.* 2004). We have also assumed a relatively low rate of species immigration. Future research should examine differences between stable and transient states in the presence of dominance competition and higher immigration rates to investigate the robustness of our findings. More generally, we have modelled plant communities in this study because much recent work on alternative states has focused on plants. Our model should be applicable to some of the animal communities characterised by dispersing larvae and sedentary adults, such as those found in intertidal habitats. However, to investigate alternative transient states more fully, future research should also use other models specifically tailored to different types of communities.

### Effects on both communities and ecosystems

The concept of alternative stable states has been used extensively for understanding not only the dynamics of community assembly, but also those of critical transitions and hysteresis affecting ecosystem-level properties (Beisner *et al.* 2003; Suding & Hobbs 2009). Critical transitions and hysteresis happen when a gradual change in environmental conditions results in a rapid and sometimes irreversible change in ecosystem states (Scheffer *et al.* 2001, 2009). Our main focus in this paper has been explanation of community structure when environmental conditions are constant over time, except for abrupt disturbance events that initiate a new round of community assembly. However, historical contingency in community assembly can affect both community- and ecosystem-level properties (Fukami *et al.* 2010). Future research should investigate the implications of alternative transient states not just for community assembly, but also for ecosystem dynamics in light of critical transitions and hysteresis (Van Geest *et al.* 2007). In doing so, it is important to also consider the effect of disturbance frequency and magnitude on transient community states (Pickett & White 1985), which we did not explicitly examine in this paper.

## Conclusion

The alternative stable states concept has greatly contributed to improving our understanding of the role of historical contingency in community assembly. However, uncritical applications of the concept may have misled us in understanding communities because, as we have shown here, the assumptions necessary to make alternative stable states relevant to explanation of transient states can be easily violated. Our results argue for a conceptual shift of attention from a narrow focus on alternative stable states to a more inclusive focus on both alternative stable states and alternative transient states. Specifically, rather than studying determinants of final variability in species composition (e.g. feedback strength, *f*), it will be more informative to also investigate those of initial variability (e.g. habitat heterogeneity, *h*) and the rate at which the initial level of variability tends toward the final level (e.g. mortality rate, *m*). We believe these efforts will allow ecologists to more tightly integrate two closely related, but historically separated subfields of community ecology – community-assembly research, which has focused on final states, and succession research, which has focused on temporal changes – for a better understanding of the influence of historical contingency in community assembly and its consequences for species diversity and ecosystem functioning.

## SUPPORTING INFORMATION

The following Supporting Information is available for this article:

**Figure S1** Temporal changes in mean colonisation rate and extinction rate.

**Figure S2** Alpha, beta and gamma diversity under different values of *h*, *f* and *m*.

**Figure S3** Same as Fig. S2, except that species diversity is measured with only species having at least 101 individuals considered a community member.

**Figure S4** Representative examples of community assembly under scenarios 1 and 2 (Fig. 1).

**Figure S5** Representative examples of community assembly under scenarios 3 and 4 (Fig. 2).

**Figure S6** Representative examples of community assembly under scenarios 5 and 6 (Fig. 3).

**Figure S7** Representative examples of community assembly under scenarios 7 and 8 (Fig. 4).

**Figure S8** Same as Fig. S2, except that results of additional simulations in which all 10 replicate communities are assembled using the same immigration history are added to each panel.

**Figure S9** Summary of all possible pair-wise comparisons of the scenarios shown in Fig. S8.

As a service to our authors and readers, this journal provides supporting information supplied by the authors. Such materials are peer-reviewed and may be re-organised for online delivery, but are not copy edited or typeset. Technical support issues arising from supporting information (other than missing files) should be addressed to the authors.
