# Supplementary figures and images for: Community assembly: alternative stable states or alternative transient states?
Source: Ecol Lett. 2011 Oct;14(10):973–84. doi: 10.1111/j.1461-0248.2011.01663.x (PMC3187870; doi:10.1111/j.1461-0248.2011.01663.x)

**Fig. S1**

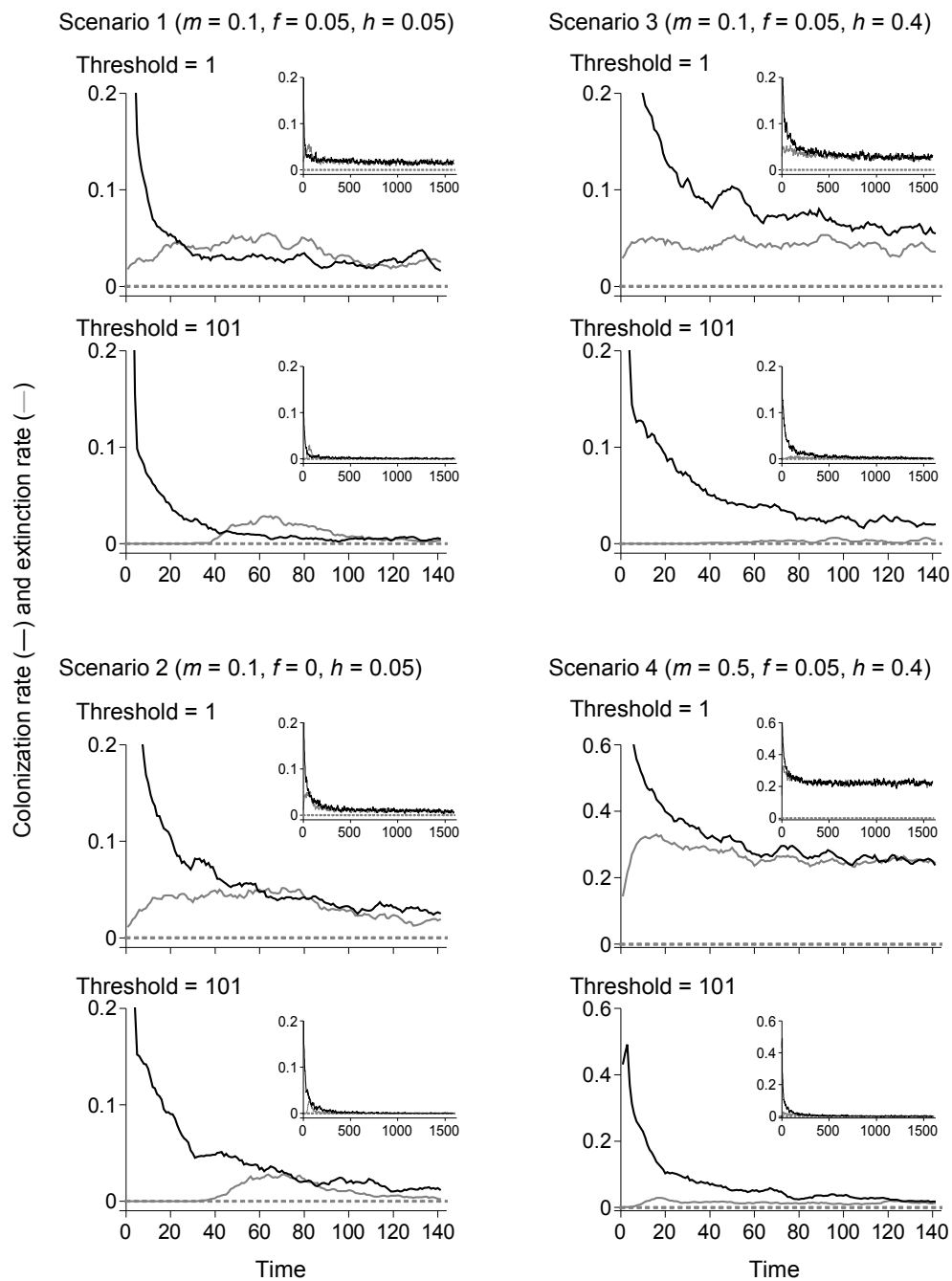

**Fig. S1 (continued)**

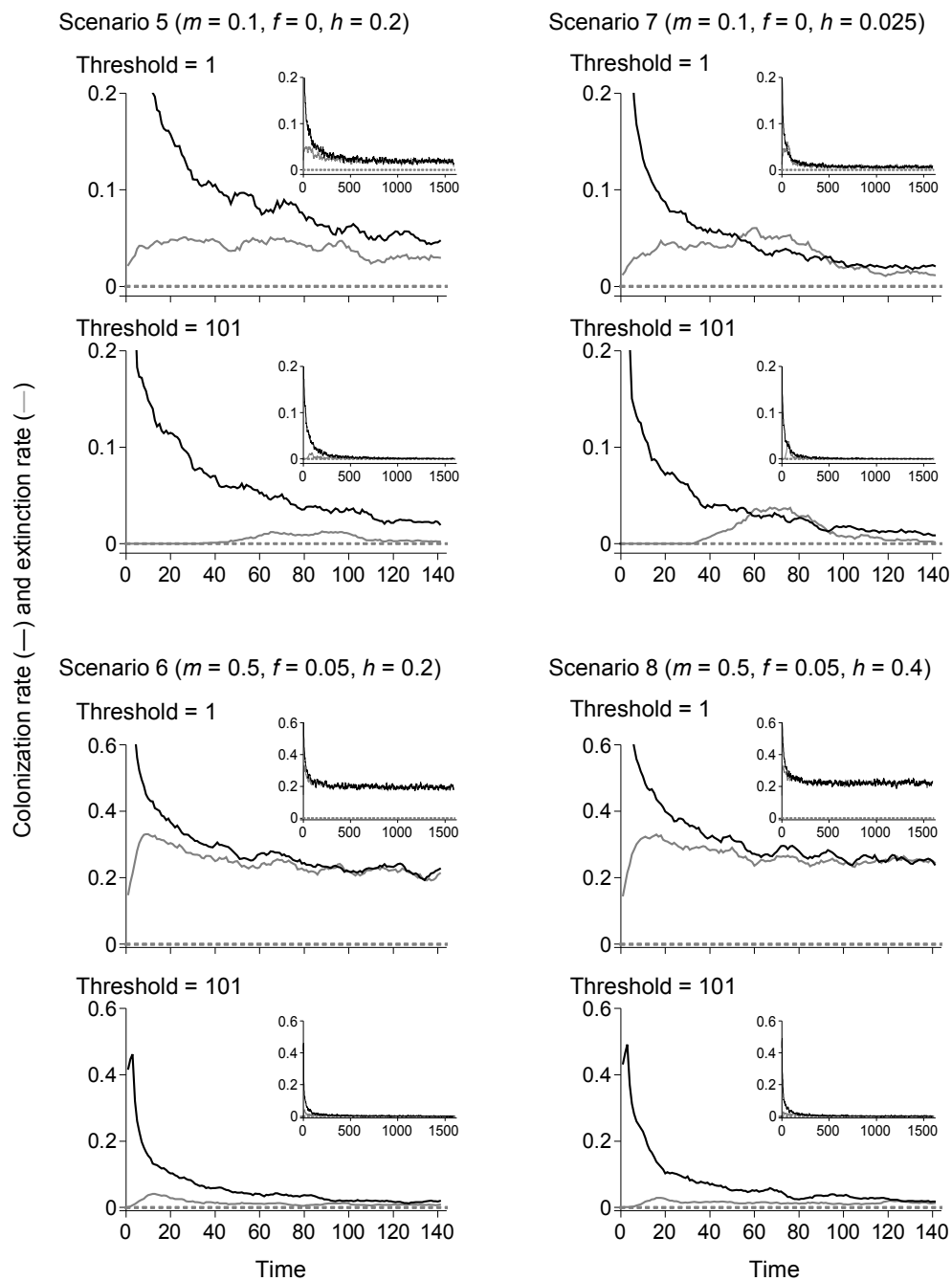

Supplement: Supplementary file 1 [file ele0014-0973-SD1.pdf]

Fig. S2

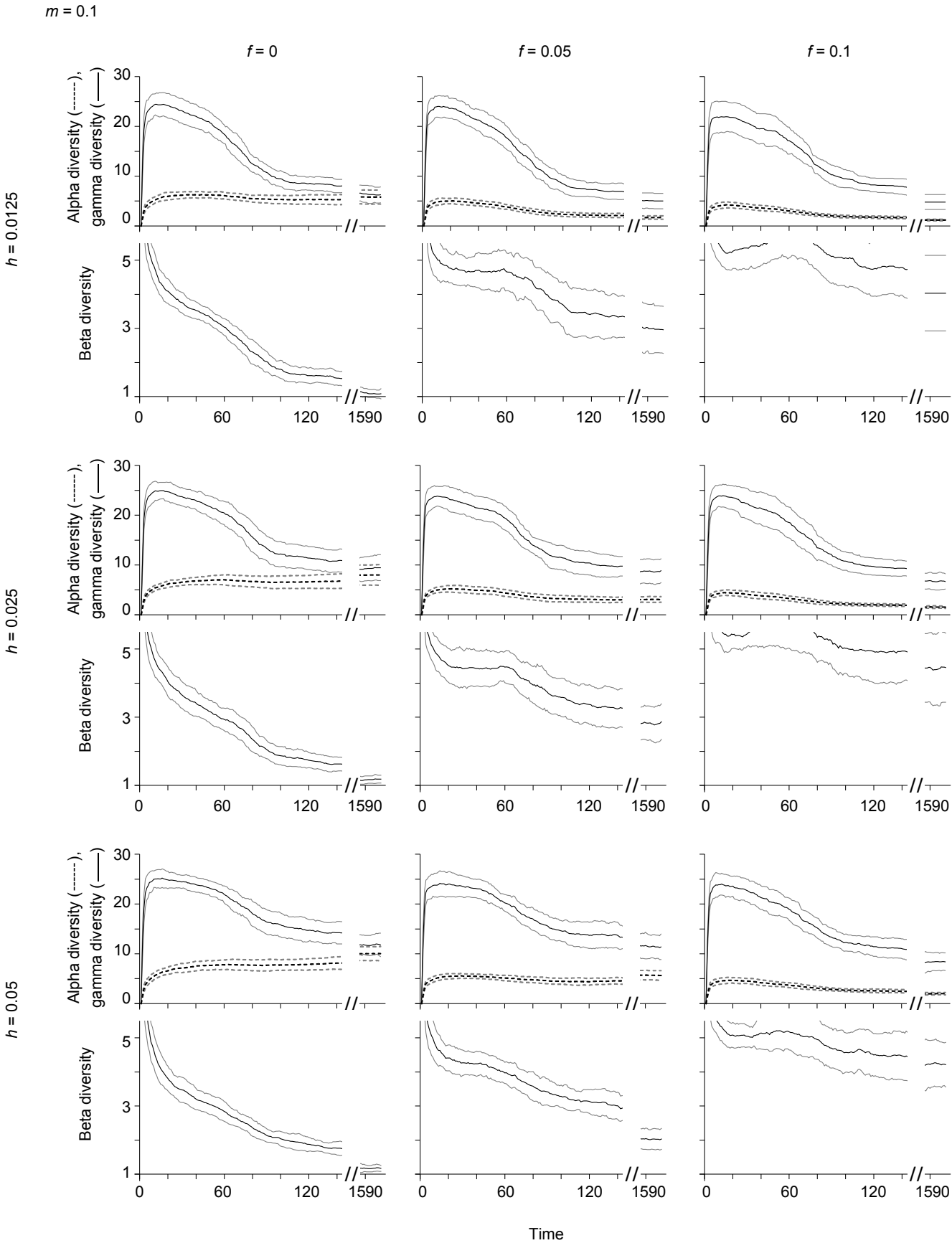

Fig. S2 (continued)

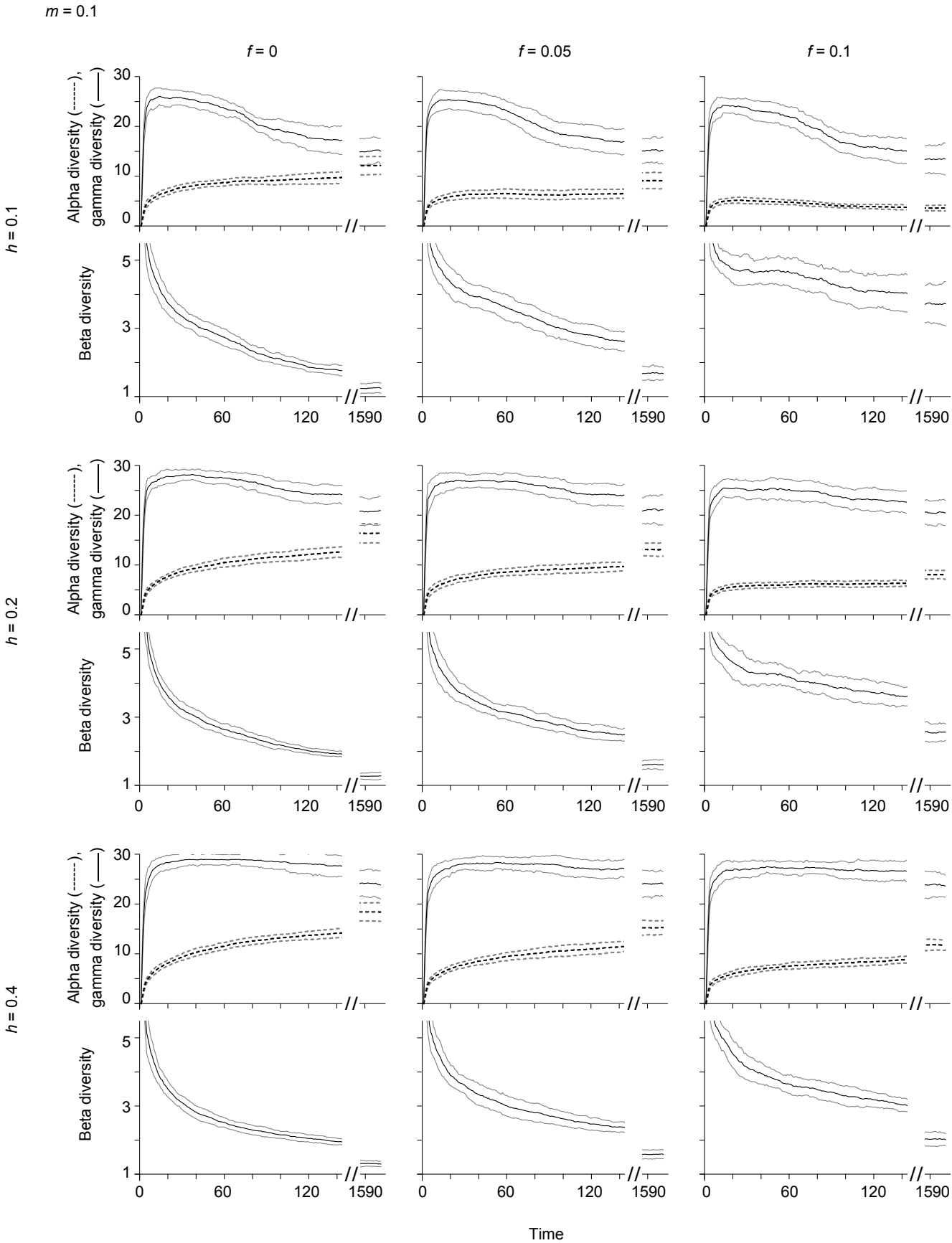

Fig. S2 (continued)

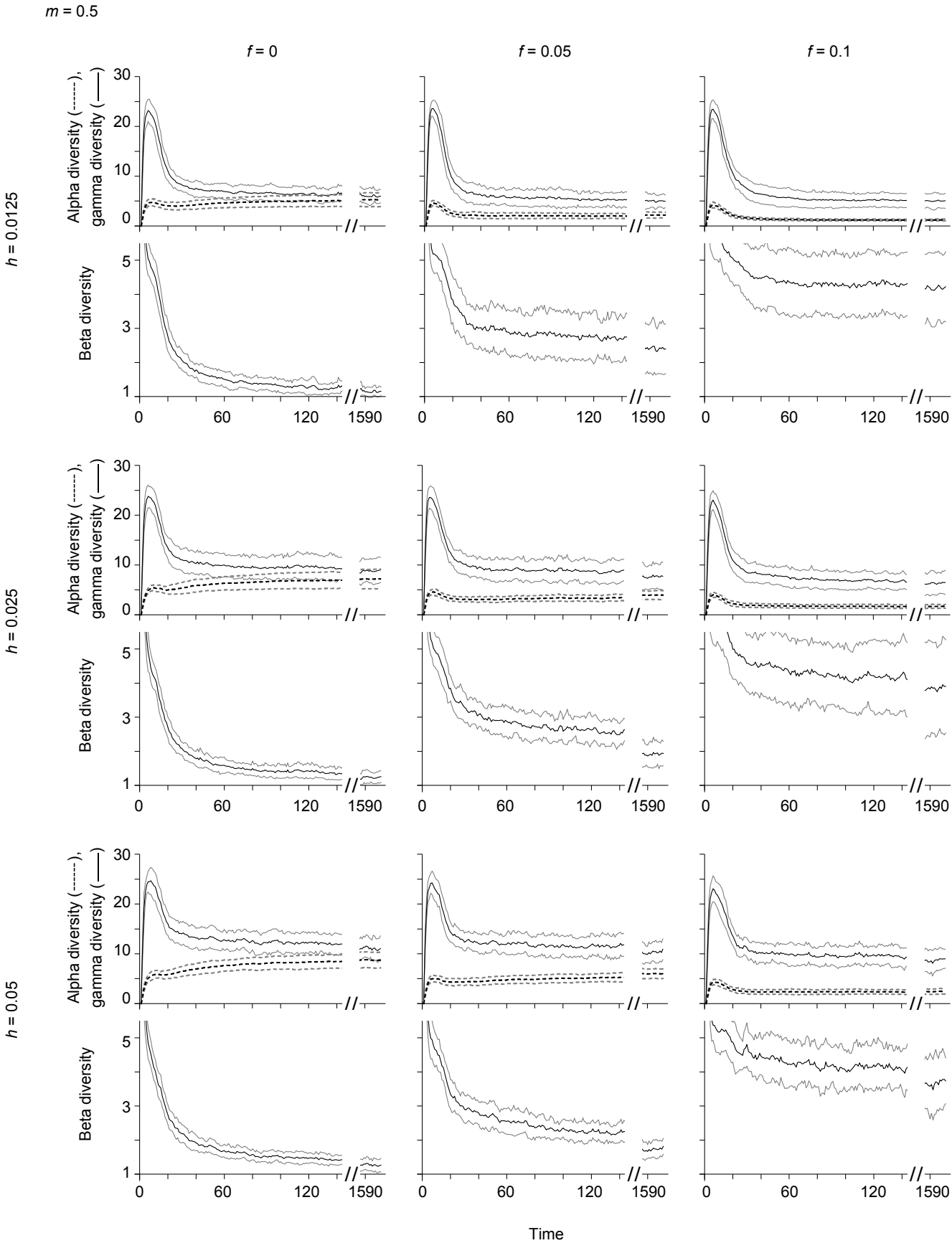

Fig. S2 (continued)

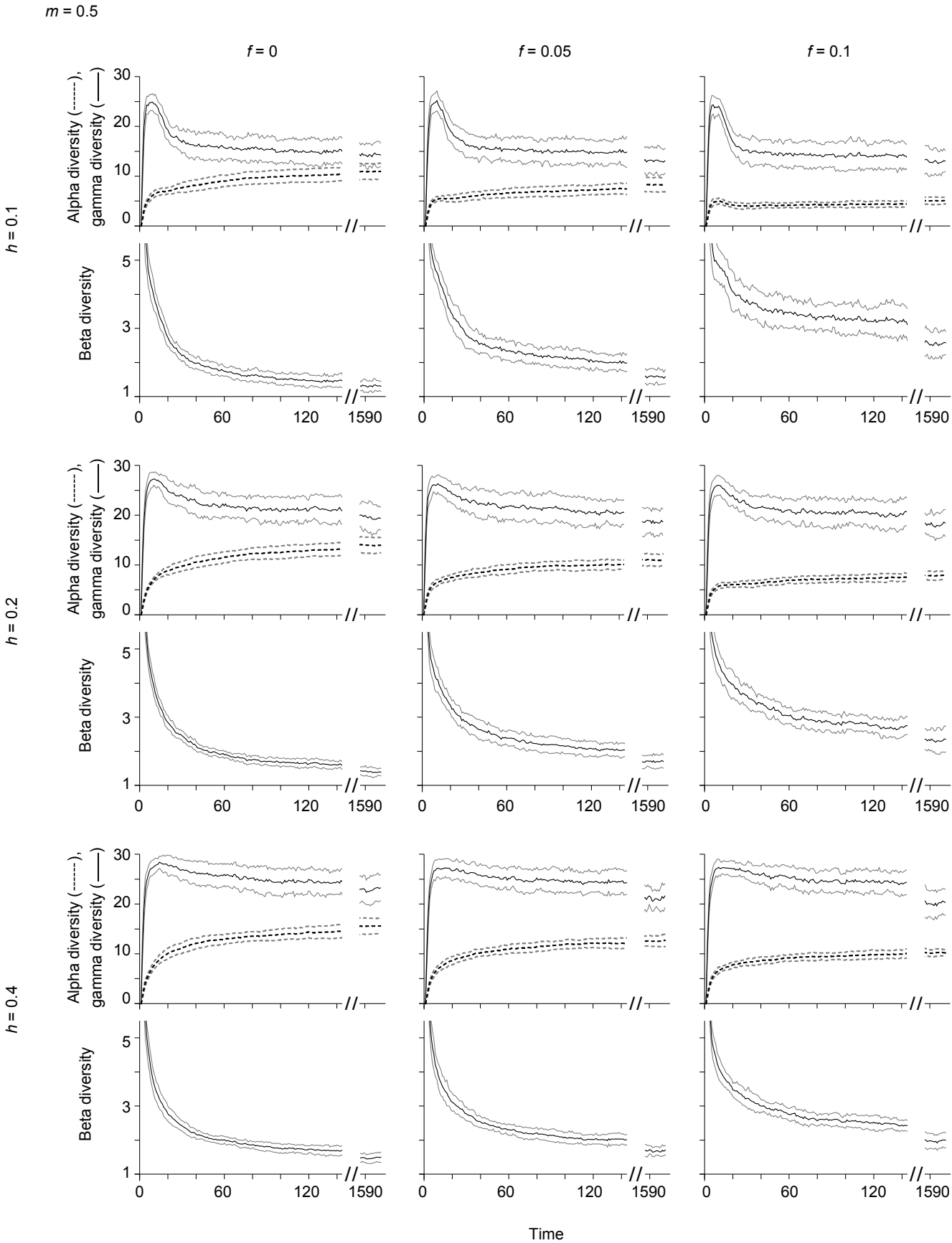

Supplement: Supplementary file 2 [file ele0014-0973-SD2.pdf]

Fig. S3

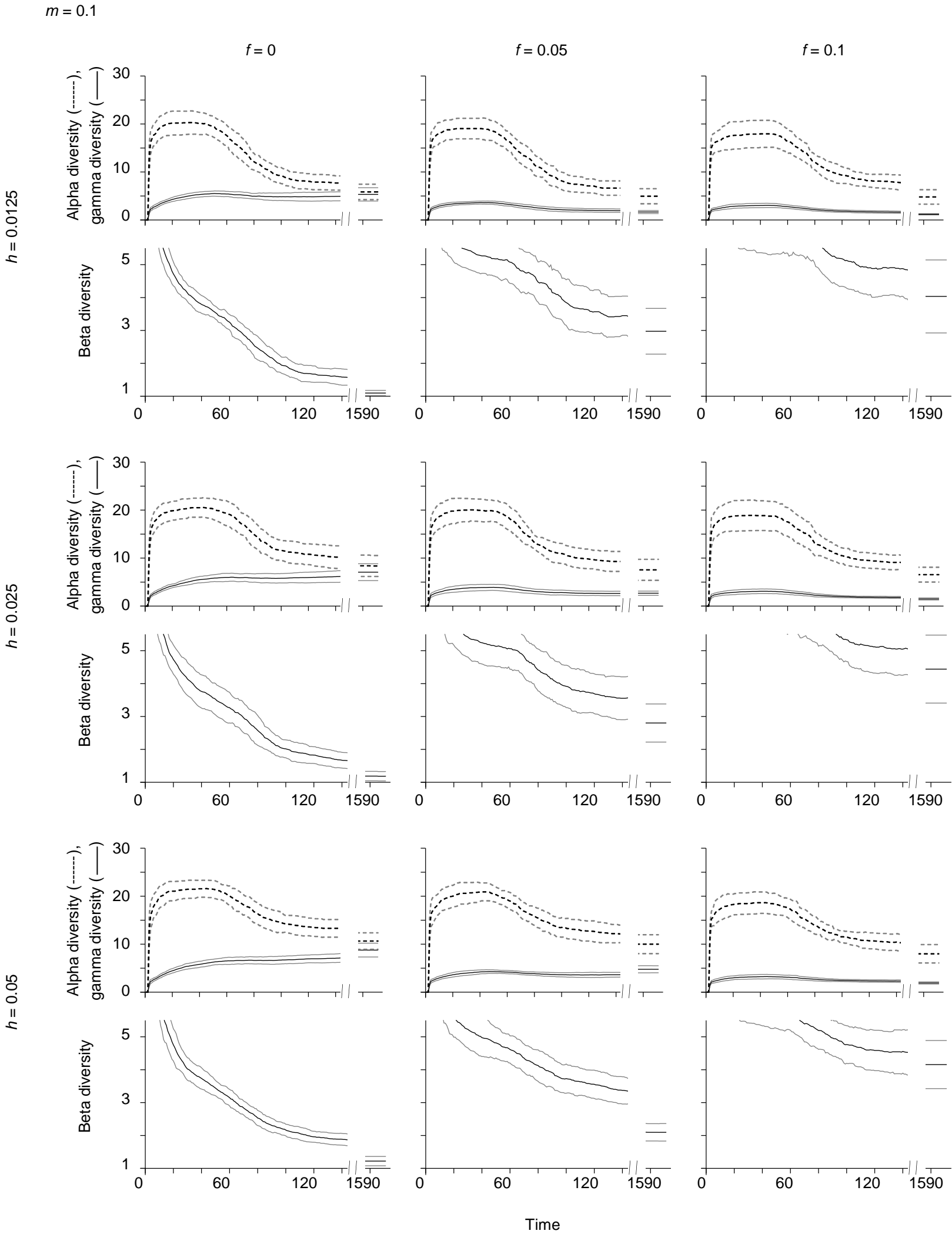

Fig. S3 (continued)

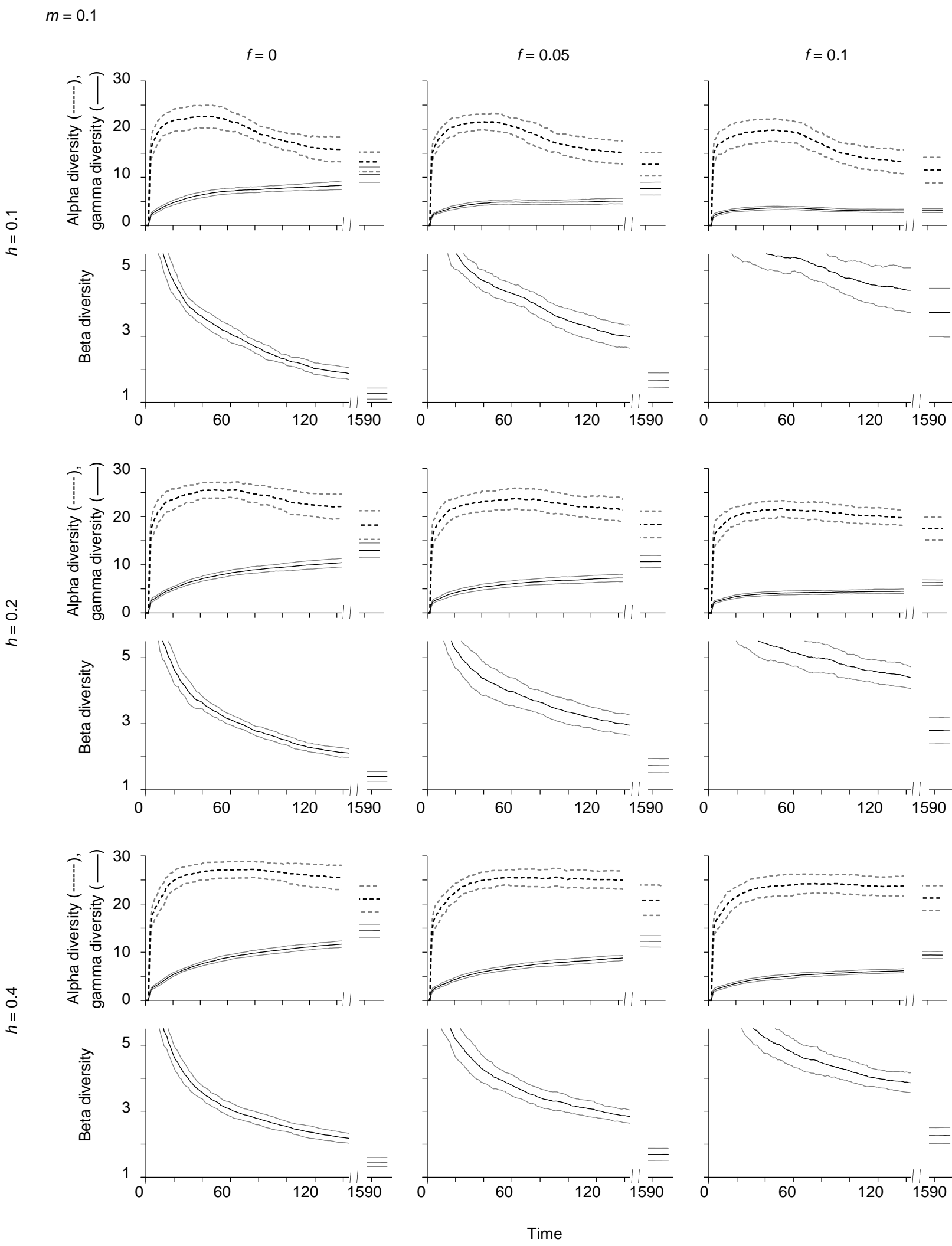

Fig. S3 (continued)

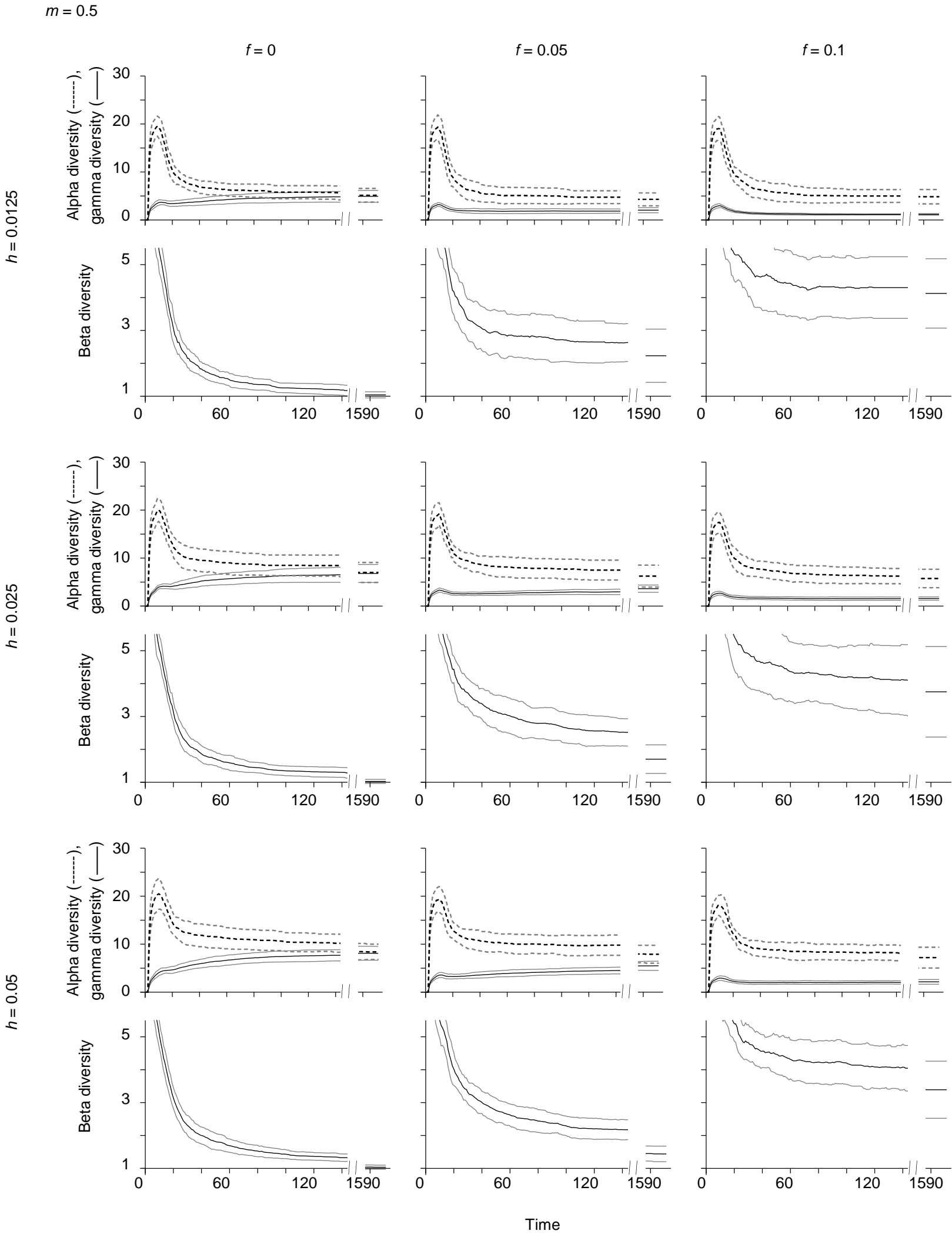

Fig. S3 (continued)

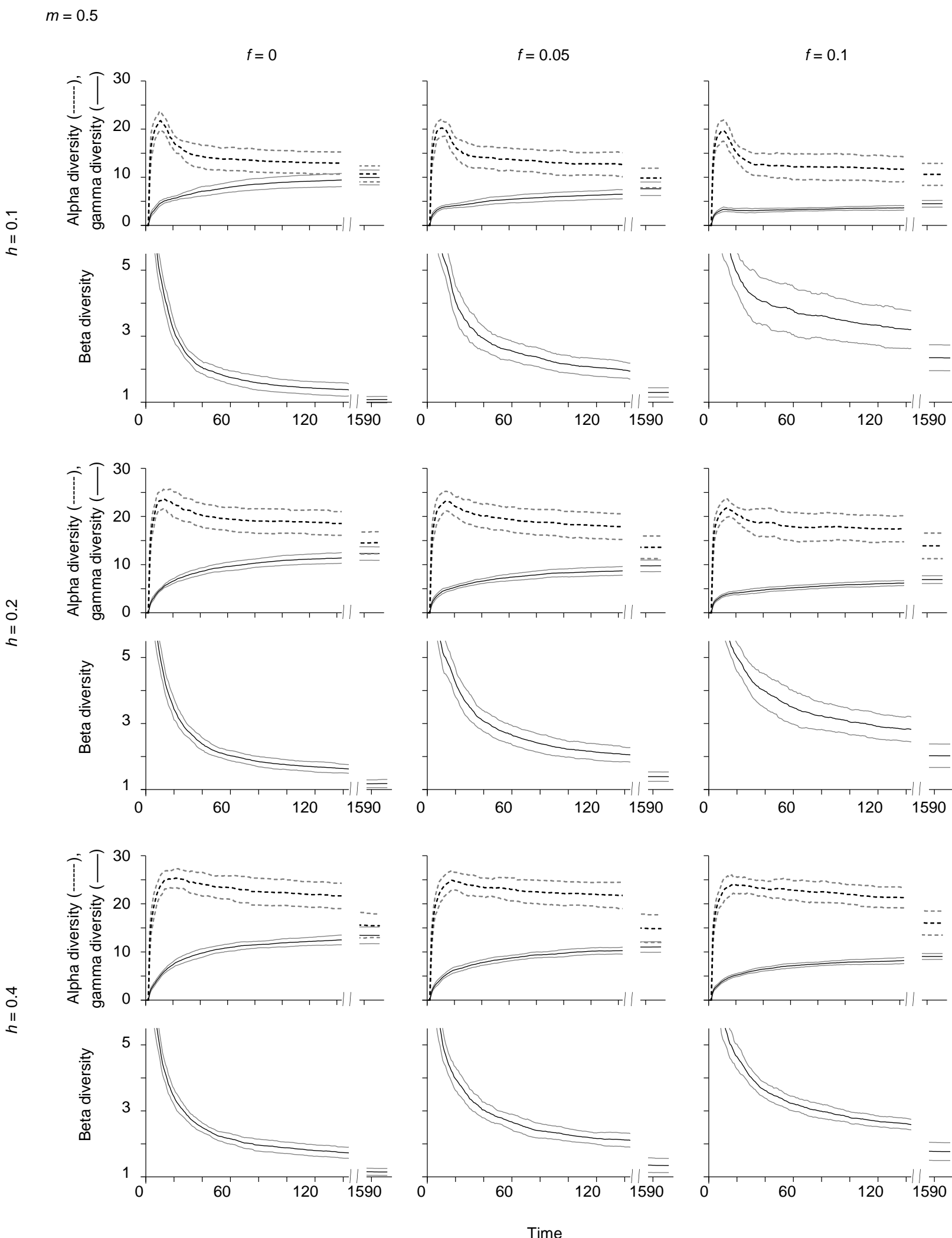

Supplement: Supplementary file 3 [file ele0014-0973-SD3.pdf]

**Fig. S4**

**(a) Scenario 1** ( $m = 0.1, f = 0.05, h = 0.05$ )

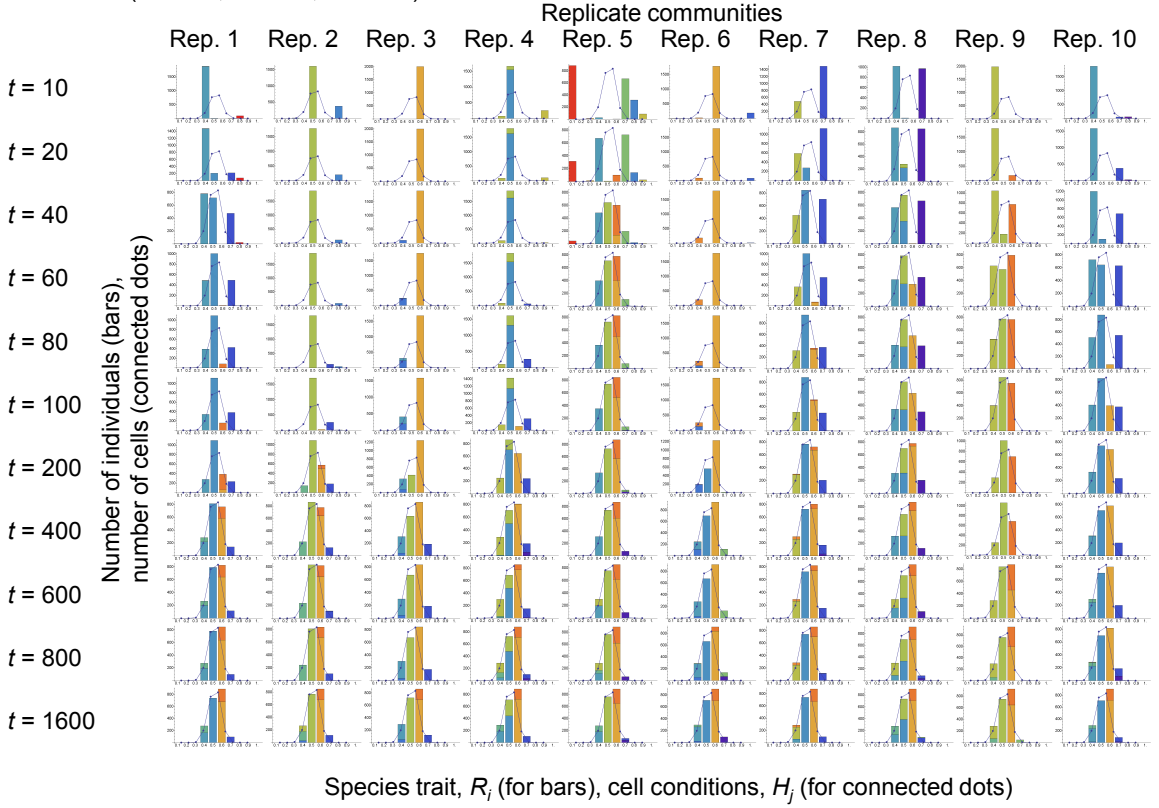

**(b) Scenario 2** ( $m = 0.1, f = 0, h = 0.05$ )

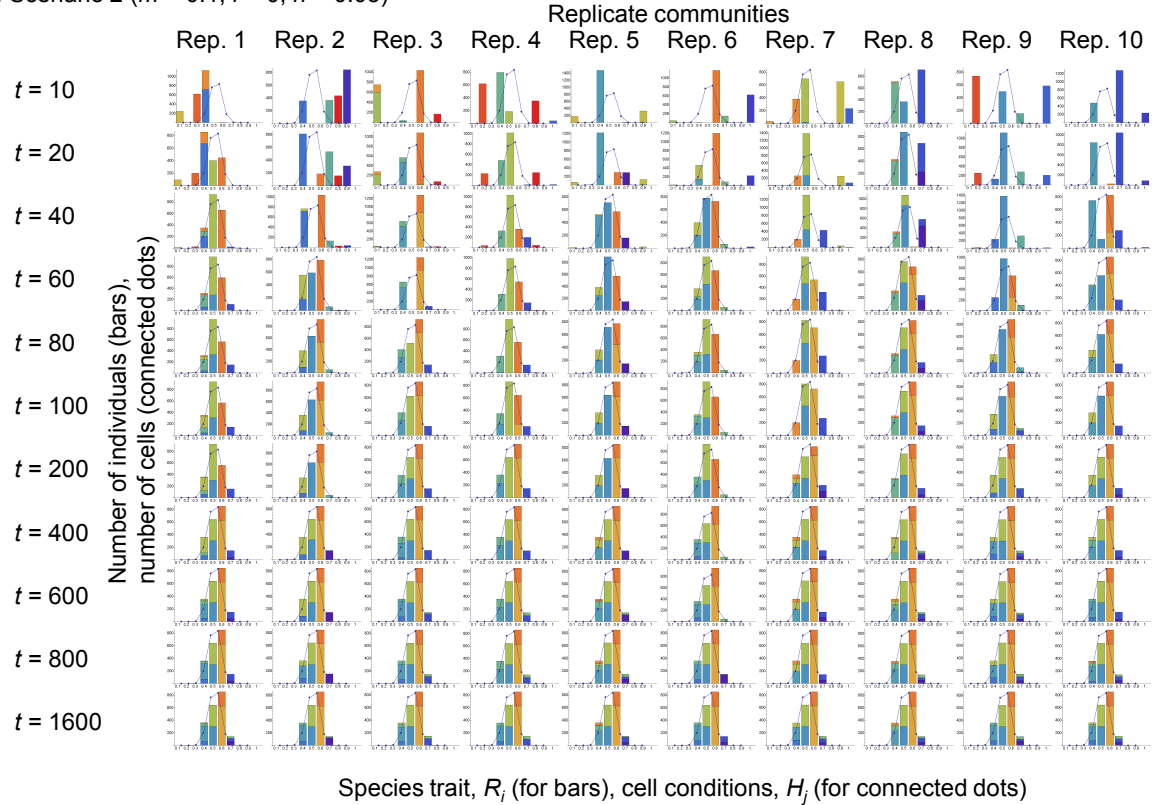

Supplement: Supplementary file 4 [file ele0014-0973-SD4.pdf]

**Fig. S5****(a) Scenario 3** ( $m = 0.1$ ,  $f = 0.05$ ,  $h = 0.4$ )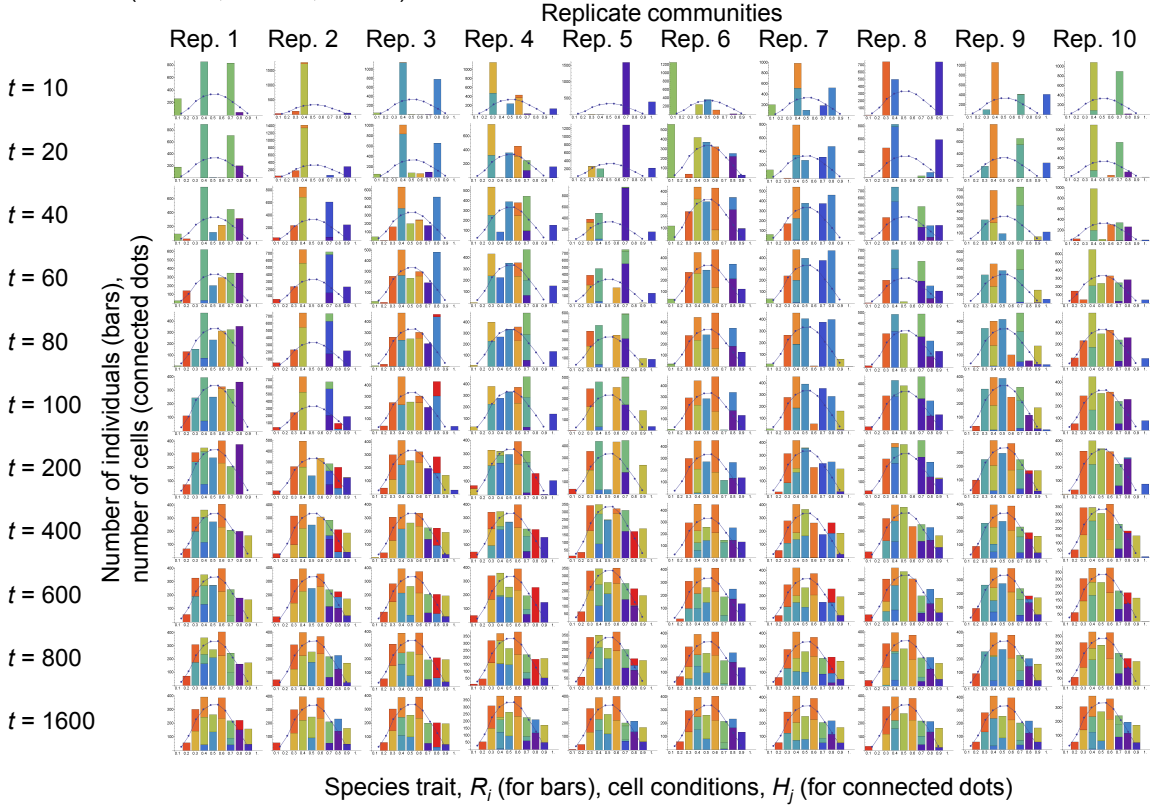**(b) Scenario 4** ( $m = 0.5$ ,  $f = 0.05$ ,  $h = 0.4$ )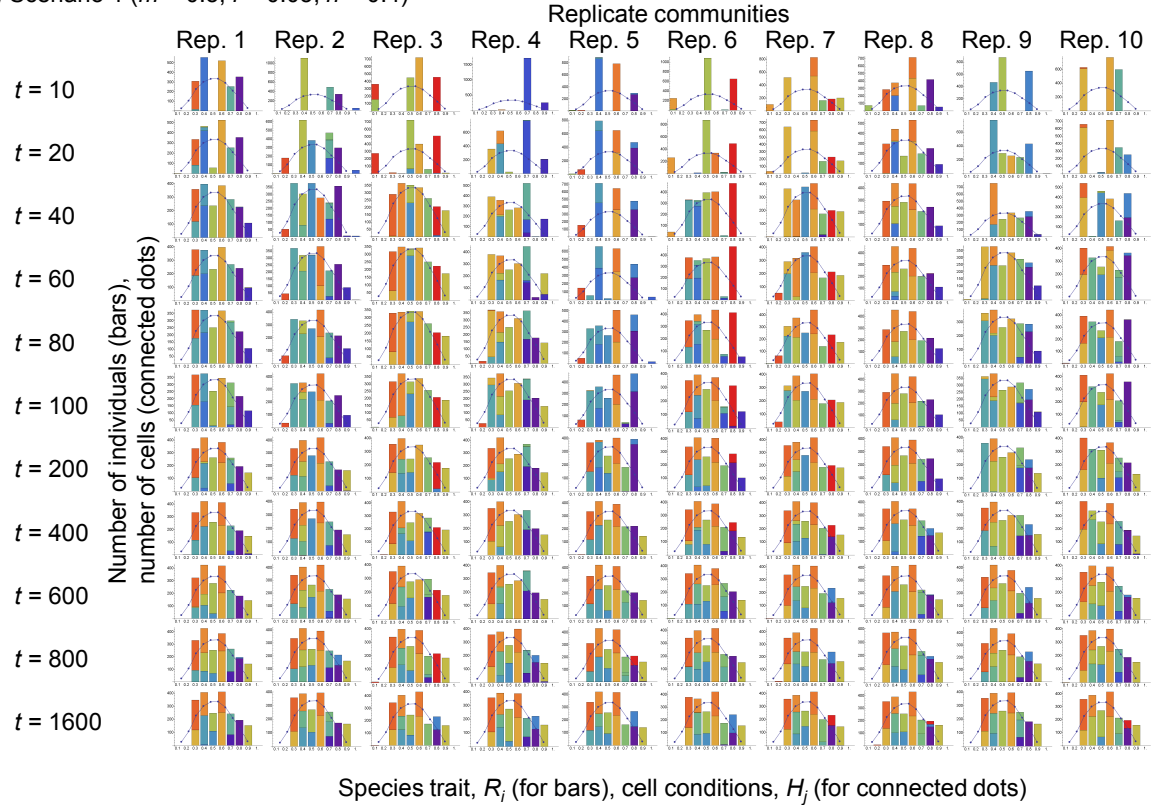

Supplement: Supplementary file 5 [file ele0014-0973-SD5.pdf]

**Fig. S6****(a) Scenario 5** ( $m = 0.1, f = 0, h = 0.2$ )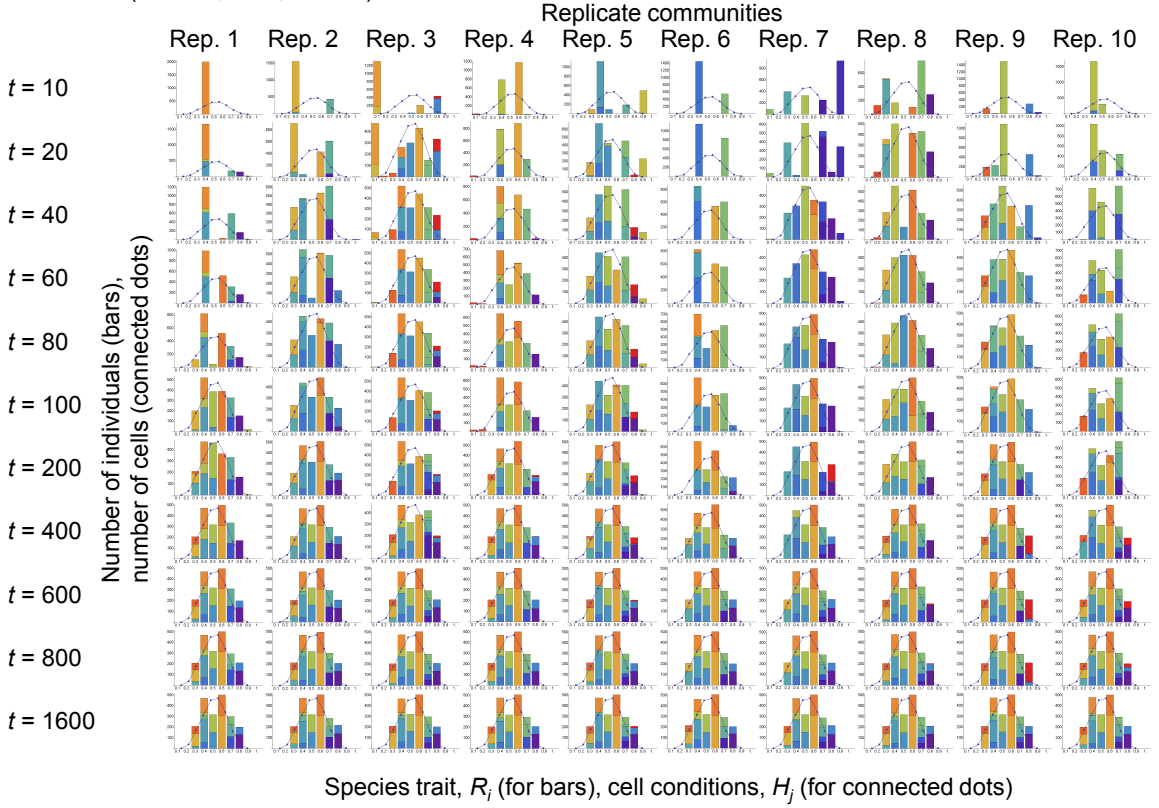**(b) Scenario 6** ( $m = 0.5, f = 0.05, h = 0.2$ )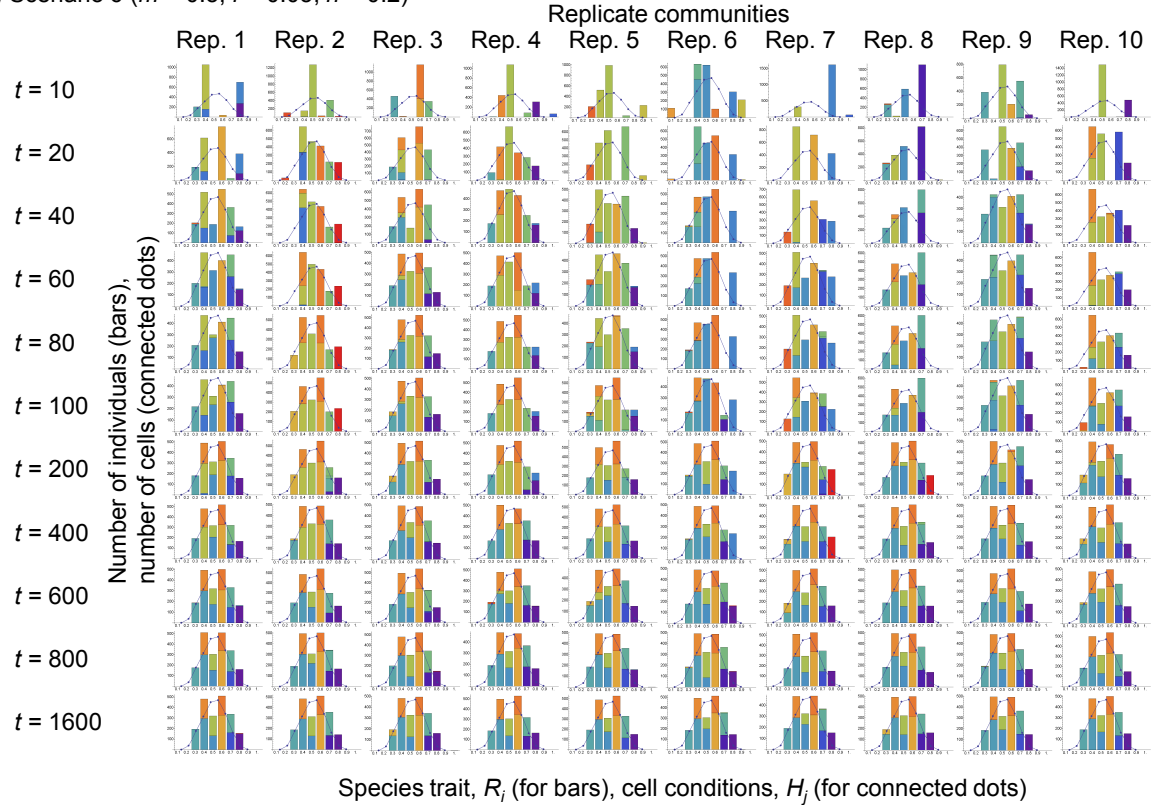

Supplement: Supplementary file 6 [file ele0014-0973-SD6.pdf]

**Fig. S7****(a) Scenario 7** ( $m = 0.1, f = 0, h = 0.025$ )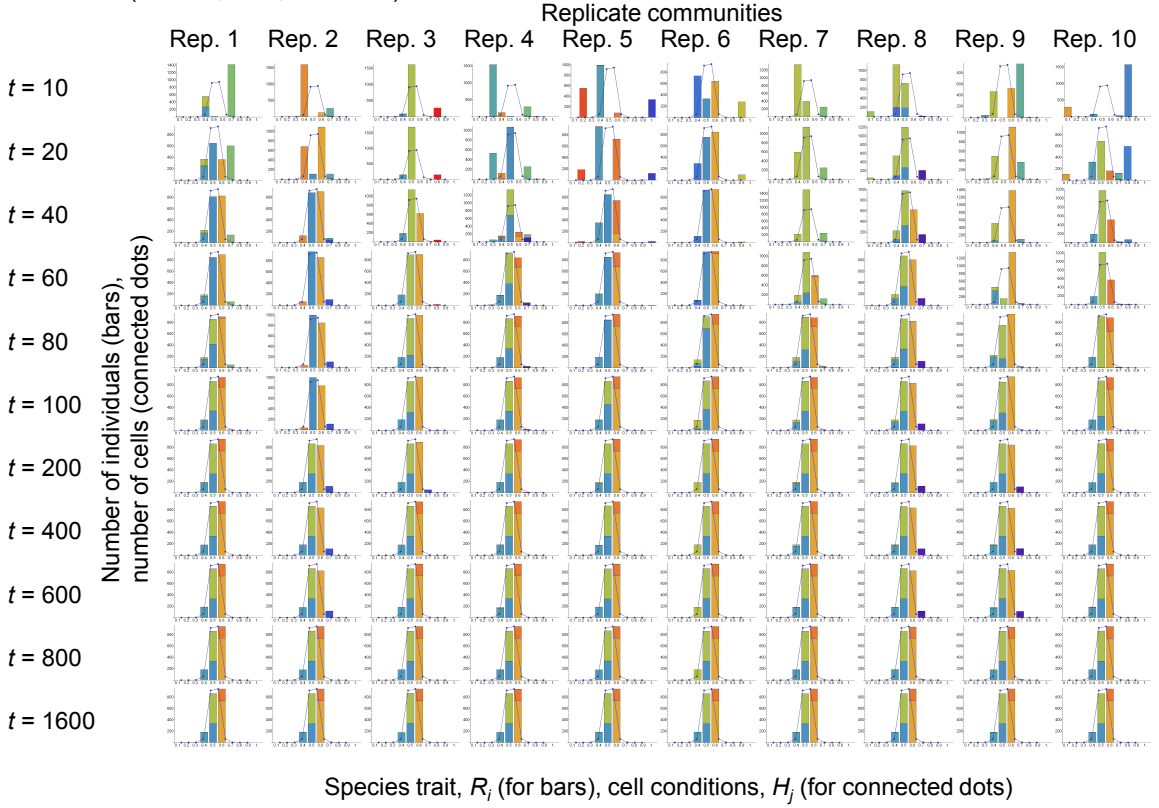**(b) Scenario 8** ( $m = 0.5, f = 0.05, h = 0.4$ )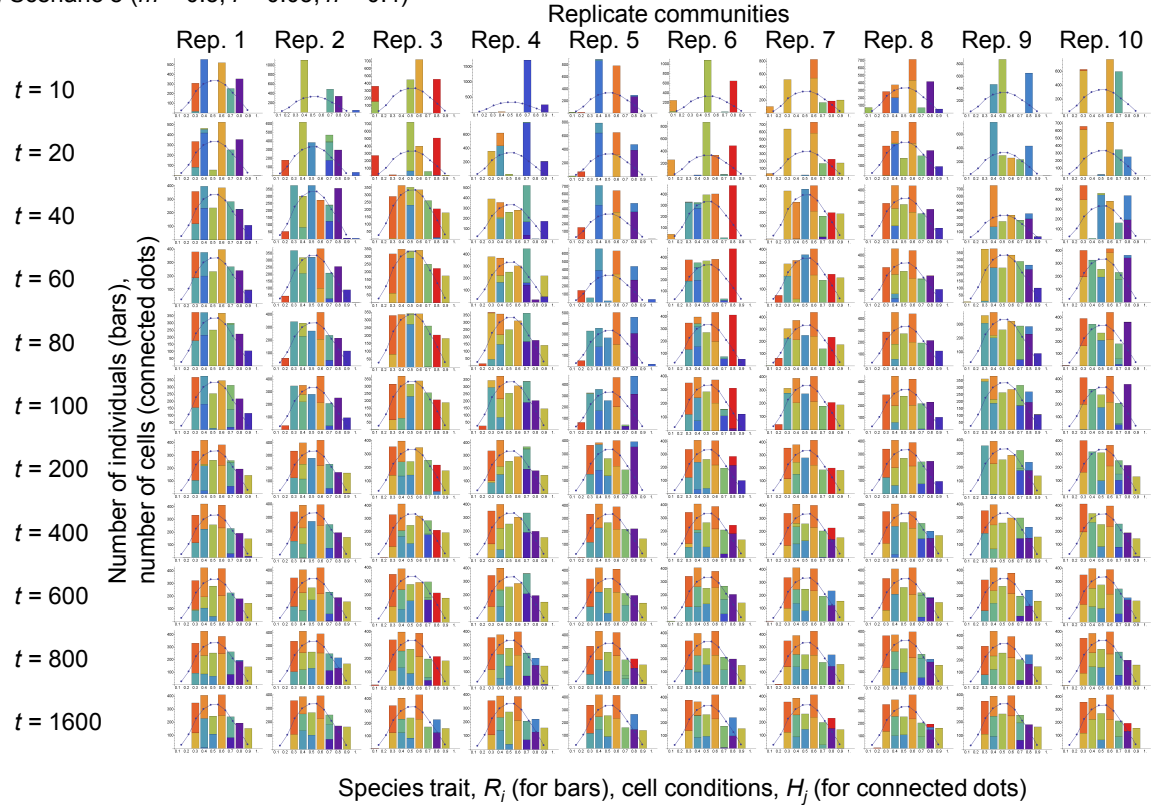

Supplement: Supplementary file 7 [file ele0014-0973-SD7.pdf]

Fig. S8

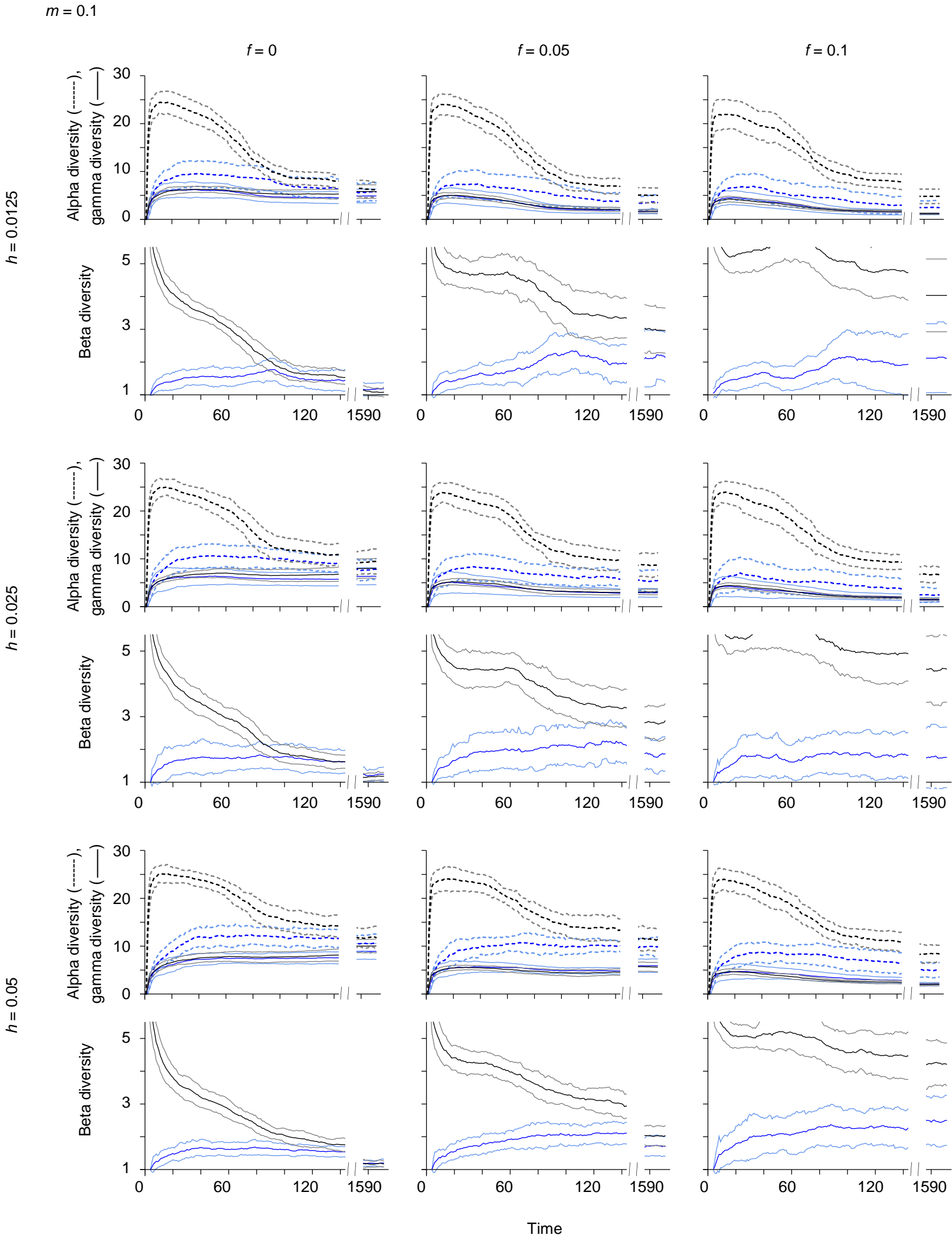

Fig. S8 (continued)

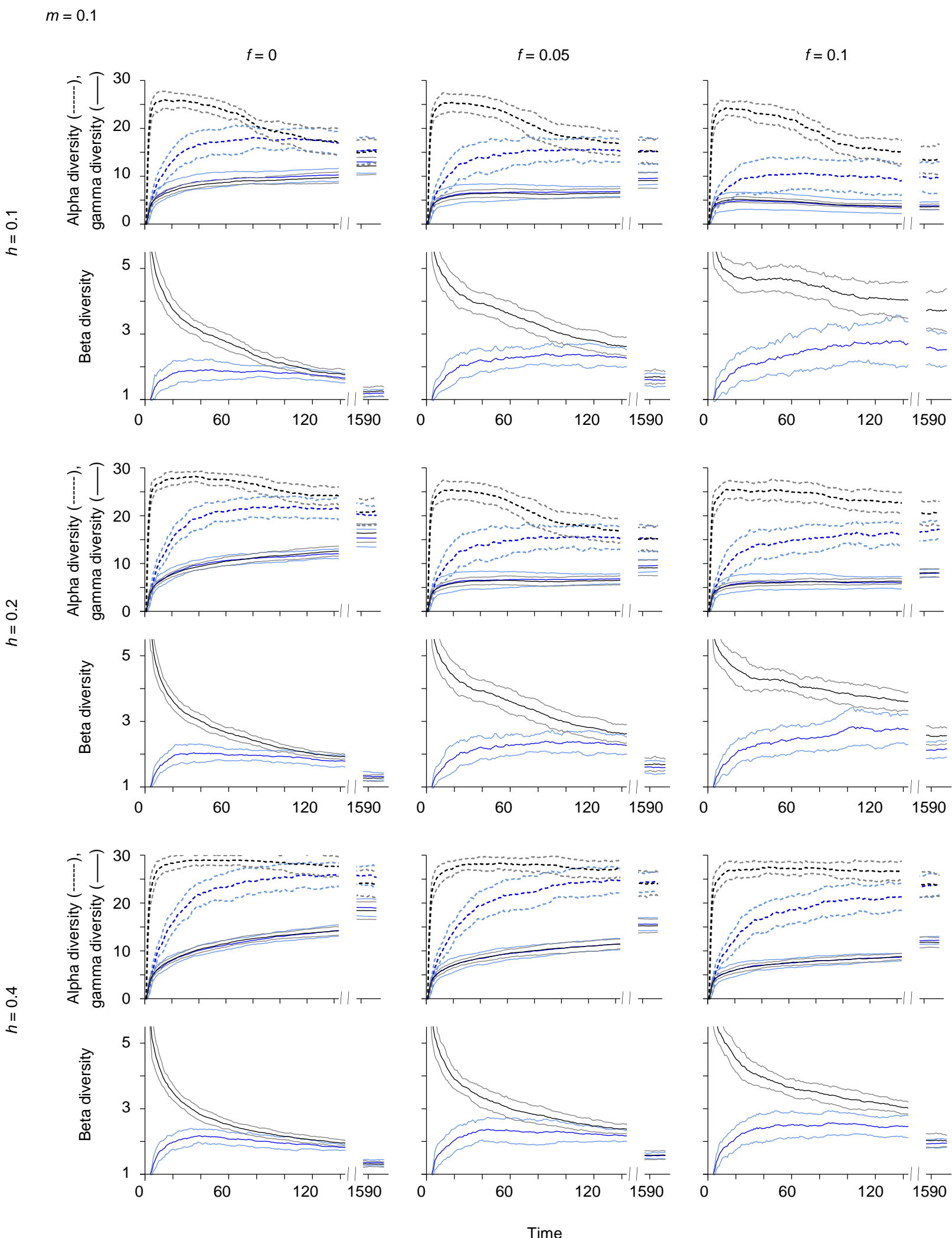

**Fig. S8 (continued)**

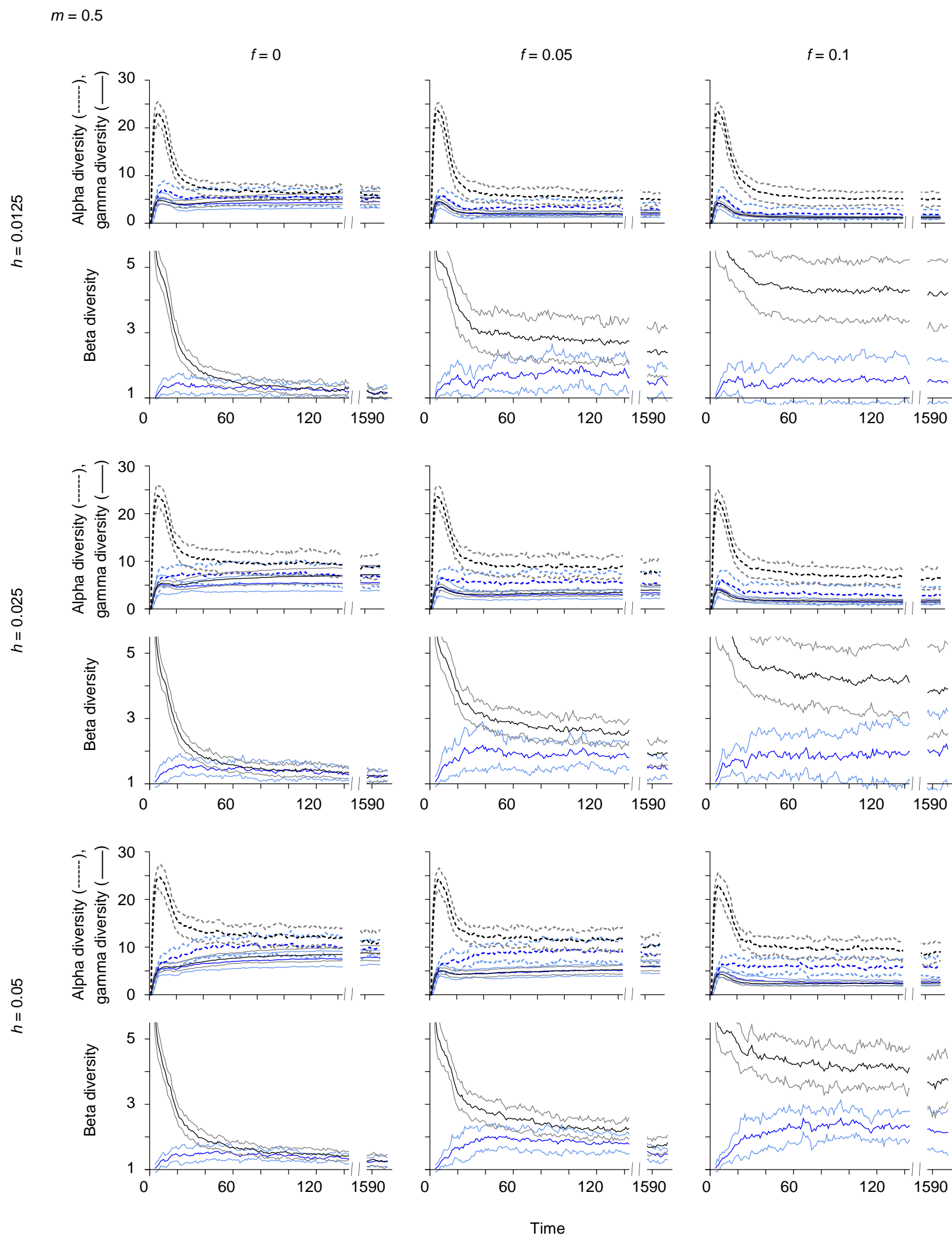

**Fig. S8 (continued)**

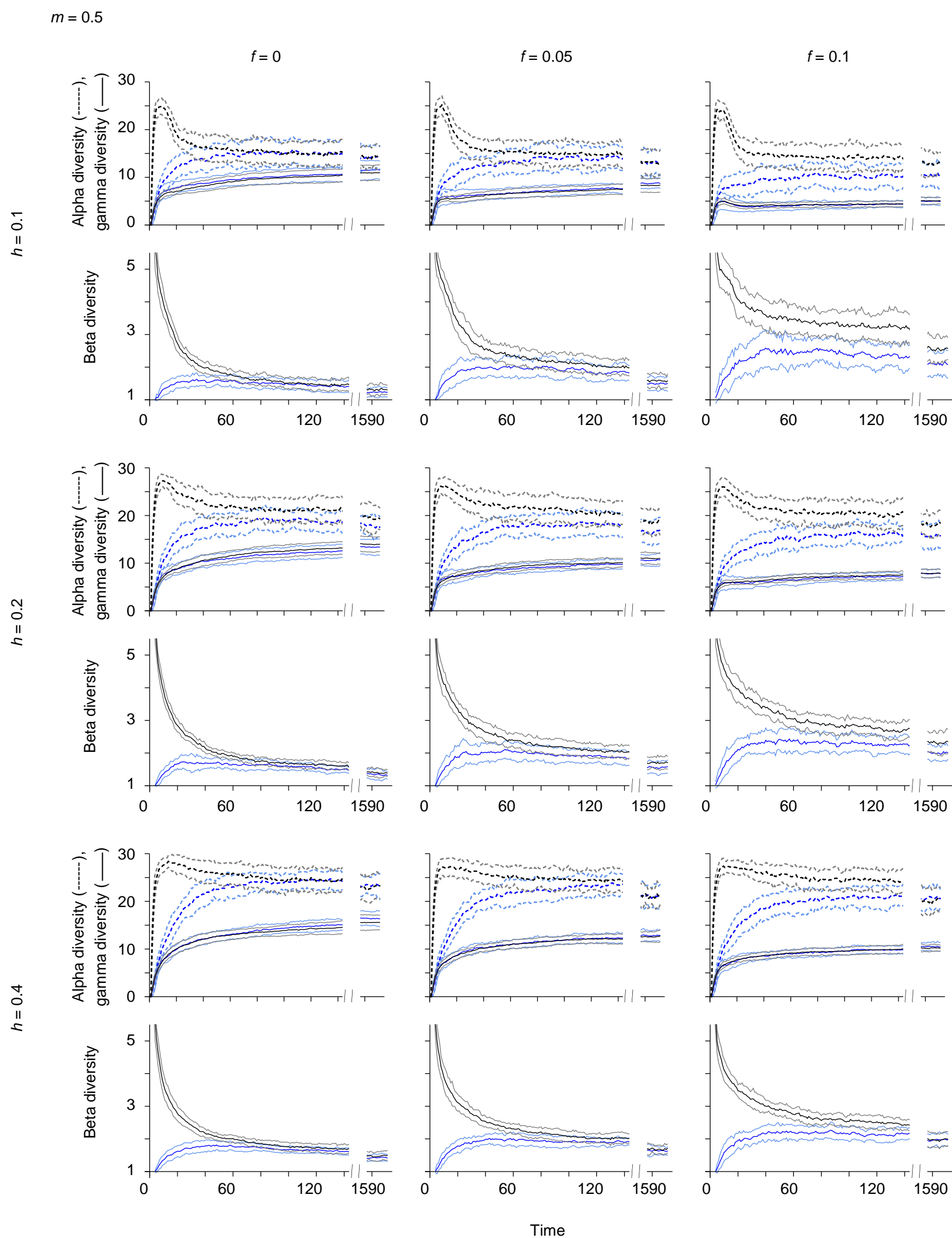

Supplement: Supplementary file 8 [file ele0014-0973-SD8.pdf]

**Fig. S9**

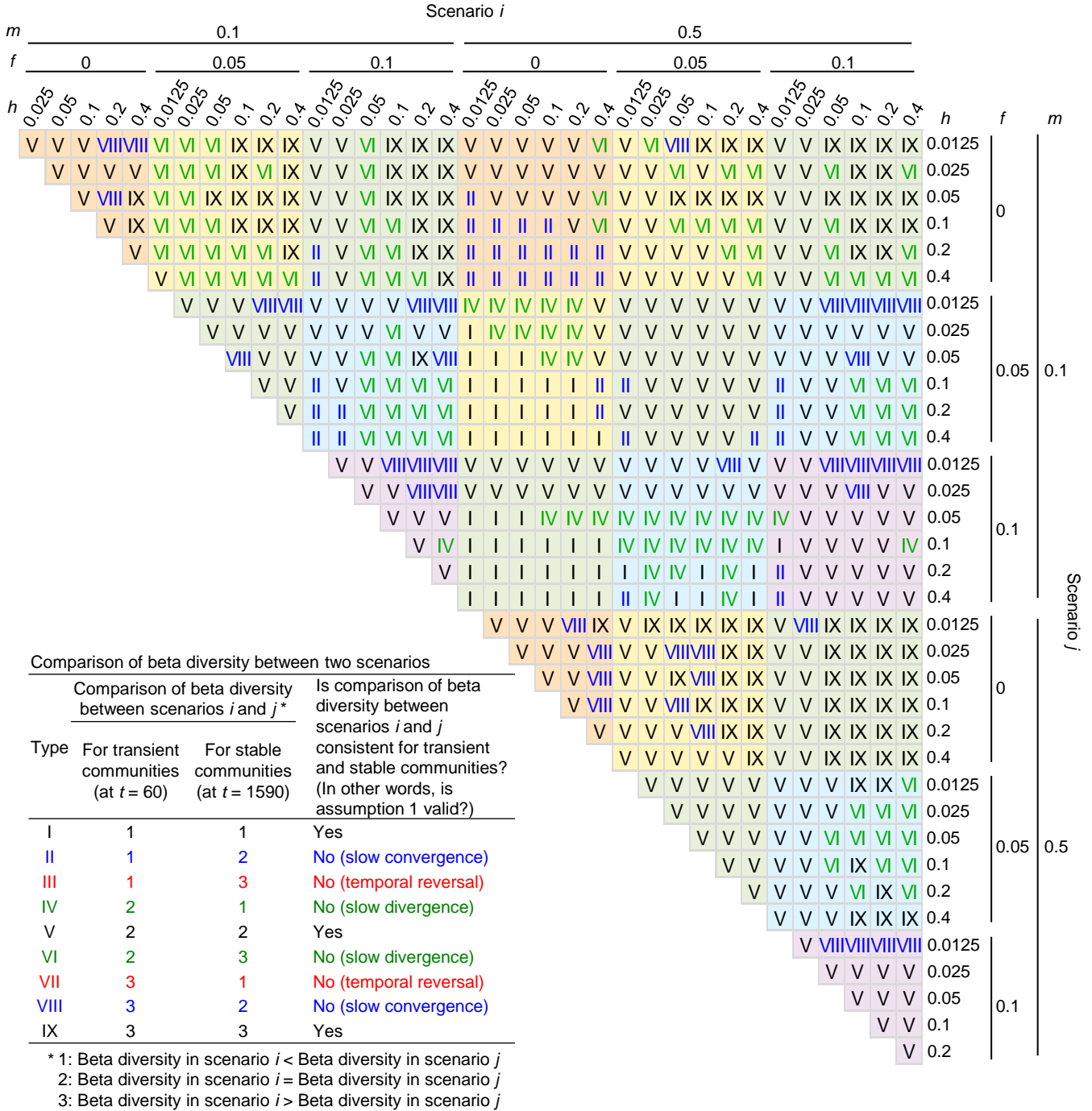

Supplement: Supplementary file 9 [file ele0014-0973-SD9.pdf]
